# STING inhibition alleviates experimental peritoneal damage: potential therapeutic relevance for peritoneal dialysis

**DOI:** 10.1002/path.6462

**Published:** 2025-08-14

**Authors:** Vanessa Marchant, Jorge García‐Giménez, Guadalupe T González‐Mateo, Pilar Sandoval, Lucía Tejedor‐Santamaria, Sandra Rayego‐Mateos, Ricardo Ramos, José A Jiménez‐Heffernan, Alberto Ortiz, Anne‐Catherine Raby, Manuel López‐Cabrera, Adrián M Ramos, Marta Ruiz‐Ortega

**Affiliations:** ^1^ Cellular and Molecular Biology in Renal and Vascular Pathology Laboratory Health Research Institute‐Fundación Jiménez Díaz University Hospital, Universidad Autónoma de Madrid (IIS‐FJD, UAM) Madrid Spain; ^2^ RICORS2040 Madrid Spain; ^3^ Laboratory of Nephrology and Hypertension Health Research Institute‐Fundación Jiménez Díaz University Hospital, Universidad Autónoma de Madrid (IIS‐FJD, UAM) Madrid Spain; ^4^ Tissue and Organ Homeostasis Program, Centro de Biología Molecular Severo Ochoa (CBM), CSIC ‐ Universidad Autónoma de Madrid Madrid Spain; ^5^ Premium Research, S.L. Guadalajara Spain; ^6^ IMDEA Food Institute, Universidad Autónoma de Madrid Madrid Spain; ^7^ Servicio de Anatomía Patológica, Hospital de la Princesa Madrid Spain; ^8^ Wales Kidney Research Unit, Division of Infection & Immunity School of Medicine, Cardiff University Cardiff UK

**Keywords:** peritoneal dialysis, peritoneal damage, peritoneal adhesions, inflammation, fibrosis, peritonitis, cytosolic DNA‐sensing pathway, STING signaling

## Abstract

Peritoneal dialysis (PD) is a widely used kidney replacement therapy for patients with end‐stage kidney disease. Nevertheless, long‐term exposure to PD fluid can damage the peritoneal membrane, leading to ultrafiltration failure and, ultimately, discontinuation of PD. Investigation of the molecular mechanisms underlying this damage is essential for identifying new therapeutic targets to mitigate peritoneal deterioration in PD patients. To this end, we employed RNA sequencing in a preclinical model of peritoneal injury, induced by prolonged chlorhexidine (CHX) exposure, which revealed cytosolic DNA‐sensing signaling as a novel pathway. Next, we demonstrated that key players in this pathway, such as the stimulator of interferon genes (STING) and its downstream signaling effectors (interferon regulatory factor 3, interferon‐stimulated genes, and nuclear factor‐κB signaling), were upregulated in experimental peritoneal damage. Moreover, increased STING expression was observed in human peritoneal biopsies from patients with PD. Subsequent studies in STING‐deficient mice showed reduced proinflammatory gene expression and immune cell infiltration, together with inhibited nuclear factor‐κB pathway activation at both early (10 days) and late (30 days) stages of CHX‐induced peritoneal injury. STING deficiency also reduced peritoneal membrane thickening, fibrosis, and mesothelial‐to‐mesenchymal transition (MMT)‐related changes in advanced CHX‐induced damage. Furthermore, pharmacological inhibition of STING with C‐176 attenuated CHX‐induced peritoneal inflammation. Macrophages were identified as one of the STING‐expressing cell types in the injured peritoneum. Hence, *in vitro* STING blockade in activated macrophages inhibited MMT in cultured mesothelial cells, suggesting that STING activation in this population may drive peritoneal fibrosis. Additionally, STING deficiency reduced peritoneal inflammation in *S. epidermidis*‐induced peritonitis and decreased adhesion scores in a postsurgical intra‐abdominal adhesion model. These findings identify STING as a pivotal mediator of peritoneal injury and support its potential as a novel therapeutic target to prevent PD‐associated ultrafiltration failure. © 2025 The Author(s). *The Journal of Pathology* published by John Wiley & Sons Ltd on behalf of The Pathological Society of Great Britain and Ireland.

## Introduction

Chronic kidney disease (CKD) is a growing global health concern, affecting over 850 million people [1] expected to become the fifth leading cause of mortality [2]. Although current therapeutic strategies may slow CKD progression, many patients progress to end‐stage kidney disease (ESKD), requiring kidney replacement therapy (KRT), and developing cardiovascular complications and other comorbidities [3, 4]. Peritoneal dialysis (PD) is a KRT that takes advantage of the peritoneum as a semipermeable membrane for the exchange of solutes and water between the blood in the peritoneal capillaries and the peritoneal dialysis fluid (PDF) infused into the peritoneal cavity [5]. Nevertheless, repeated and prolonged exposure of the peritoneum to conventional PDFs, utilizing glucose as an osmotic agent, results in peritoneal injury that ultimately leads to ultrafiltration failure and therapy discontinuation [6]. This PDF exposure induces a series of cellular and molecular changes in the peritoneal membrane (PM), including loss of mesothelial cells (MCs) by death, detachment, or mesothelial‐to‐mesenchymal transition (MMT), and sustained inflammation [7]. The peritoneal inflammatory/immune response is characterized by macrophage and T‐cell recruitment, which is linked to the activation of different intracellular pathways, including nuclear factor (NF)‐κB signaling [8]. These pathological responses lead to submesothelial fibrosis, intra‐abdominal adhesion formation, calcification, and neovascularization [9, 10, 11, 12]. In addition, peritoneal infections, mainly bacterial, may also occur, leading to recurrent episodes of peritonitis that exacerbate both local and systemic damage [13]. Despite extensive research, a comprehensive understanding of the molecular mechanisms underlying these harmful processes remains elusive.

Activation of innate immunity is critical for the clearance of infectious agents but is also involved in the response to damage‐associated molecular patterns (DAMPs) released by stressed or damaged cells [14, 15]. Exogenous DNA from viruses or bacteria, as well as host DNA from stressed mitochondria and damaged genomic DNA released into the cytoplasm, can act as DAMPs. These are recognized by cytosolic DNA sensors that activate cell signaling cascades, such as the cyclic GMP‐AMP (cGAMP) synthase (cGAS)–stimulator of interferon genes (STING) signaling pathway [16]. Following its interaction with cytoplasmic double‐stranded DNA (dsDNA), cGAS synthesizes the second messenger, cGAMP, which is recognized by STING [17, 18]. Upon binding to cGAMP, STING translocates from the endoplasmic reticulum (ER) to the ER–Golgi intermediate compartment (ERGIC) and Golgi apparatus. During this process, STING recruits serine/threonine‐protein kinase TBK1 (TBK1), which autophosphorylates, phosphorylates STING, and subsequently phosphorylates and activates the transcription factor interferon regulatory factor 3 (IRF3), ultimately leading to induction of type I interferon (IFN) expression [19, 20]. By binding to their surface receptors, type I IFNs activate the transcription of a set of interferon‐stimulated genes (ISGs) [21, 22]. In addition to downstream activation of the IFN pathway, STING can also recruit the inhibitor of nuclear factor kappa‐B kinase subunit epsilon (IKKε) to coordinately activate the NF‐κB pathway, resulting in the transcription of a series of proinflammatory cytokines and chemokines [20, 23]. For this reason, among the components of this pathway, STING has particular relevance in immune/inflammatory disorders. STING hyperactivation has been described in auto‐inflammatory and autoimmune diseases and, more recently, in an increasing number of chronic inflammatory conditions, including cardiovascular diseases [24, 25]. Furthermore, STING modulation, specifically in endothelial cells, controls T‐cell recruitment into the peritoneal cavity in mice [26]. However, the involvement of STING in PD‐associated peritoneal damage has not yet been investigated.

Here, we report transcriptomic analysis in a preclinical model of peritoneal injury, which identified novel mediators involved in peritoneal damage and highlighted the cytosolic DNA‐sensing pathway as one of the most relevant signaling pathways involved in peritoneal damage. STING, a key player in this pathway, and its related downstream signaling were activated in response to experimental peritoneal injury of diverse etiologies (including sterile and infectious), while STING genetic deficiency or its pharmacological inhibition alleviated peritoneal injury. In support of the translational relevance of these findings, STING was found to be overexpressed in peritoneal biopsies from PD patients. Therefore, we propose STING as a novel promising therapeutic target to prevent peritoneal deterioration associated with the complications of PD treatment in ESKD patients.

## Materials and methods

### Ethics approval and patient consent

All animal procedures were performed in mice according to the European Community and Animal Research: Reporting of In Vivo Experiments (ARRIVE) reporting guidelines for the care and use of laboratory animals, with the prior approval by the Animal Ethics Committee of the IIS‐Fundación Jiménez Díaz and Comunidad Autónoma de Madrid, Spain (PROEX 242.2/21).

Experiments on peritoneal biopsies from patients were performed according to the Declaration of Helsinki guidelines [27]. Written informed consent was obtained from all patients prior to sample obtention and approved by the Ethics Committee of Hospital Universitario La Paz, Madrid, Spain (HULP PI‐4600; Ref. 07/253477.9/21).

### Human samples

Formalin‐fixed paraffin‐embedded peritoneal biopsies were used for STING immunostaining. Case biopsies were obtained from ESKD‐PD patients at the time of kidney transplantation or catheter replacement. Control biopsies were obtained from hemodialysis patients at the time of kidney transplantation or predialysis patients at the time of catheter insertion for PD. Demographic and clinical characteristics of patients are shown in supplementary material, Table S1. Data are reported following the STrengthening the Reporting of OBservational studies in Epidemiology (STROBE) guidelines and Biospecimen Reporting for Improved Study Quality (BRISQ) recommendations [28, 29].

### Animal models

C57BL/6J WT and C57BL/6J‐STING1gt/J (systemic STING deficiency; STING‐KO) male and female mice (8‐ to 14‐week‐old) were obtained from Charles River Laboratories Spain (Barcelona, Spain) and maintained at the IIS‐Fundación Jiménez Díaz animal facilities, with free access to food and water, normal light/dark cycles, and under specific pathogen‐free conditions. Several peritoneal injury models were developed in WT and STING‐KO mice: CHX exposure models, a postsurgical intra‐abdominal adhesion model, and an *S. epidermidis*‐induced peritonitis model. A 10‐day CHX exposure model was also developed in parallel by administration of the STING pharmacological inhibitor C‐176. STING immunostaining was evaluated in two chronic PDF exposure models previously established in our laboratory, involving either 5/6 nephrectomized mice (CKD model) or non‐CKD mice. In brief, Stay Safe PDF (lactate‐buffered, 4.25% glucose) (Fresenius Medical Care, Bad Homburg, Germany) was administered using a PD catheter connected to a subcutaneous mini access port for either 30 or 60 days, as previously described [30]. The mice were then euthanized, and peritoneal lavages and parietal peritoneal tissue samples were collected according to the specific requirements for subsequent histological analyses (staining, immunohistochemistry, and immunofluorescence), protein analysis (western blotting), gene expression (RT‐qPCR and RNA‐seq), and flow cytometry. Data from animal models are reported following the ARRIVE guidelines [31].

### Transcriptomic analyses

An RNA sequencing (RNA‐seq) study was carried out using peritoneal tissue samples from control and 10‐day‐CHX‐injected male mice. RNA extraction, library preparation, and sequencing on a NextSeq 2000 sequencer (Illumina, San Diego, CA, USA) were performed. Read cleaning, mapping, alignment, and differential expression analyses were subsequently carried out. Different functional enrichment analyses were then conducted using the differentially expressed genes (DEG) identified.

### Cell culture

The human MC line MeT‐5A (ATCC, Rockville, MD, USA) was used for *in vitro* assessment of MMT in response to activated macrophage‐conditioned media (AMCM). AMCM were obtained from lipopolysaccharide (LPS)‐activated primary murine peritoneal macrophages pretreated or not with the STING pharmacological inhibitor C‐176 (control AMCM and C‐176 AMCM, respectively), as previously described [32]. Following the experiments, gene expression in the MCs was evaluated.

Further details on the methodology employed for animal models, sample processing, histological staining, immunohistochemistry, immunofluorescence, western blotting, RT‐qPCR, RNA sequencing, omics data analysis, flow cytometry, statistical procedures, and reagents used, including antibodies and pre‐designed qPCR assays (supplementary material, Table S3) are provided in the Supplementary materials and methods.

## Results

### Transcriptomic changes in experimental peritoneal injury

**Figure 1:**
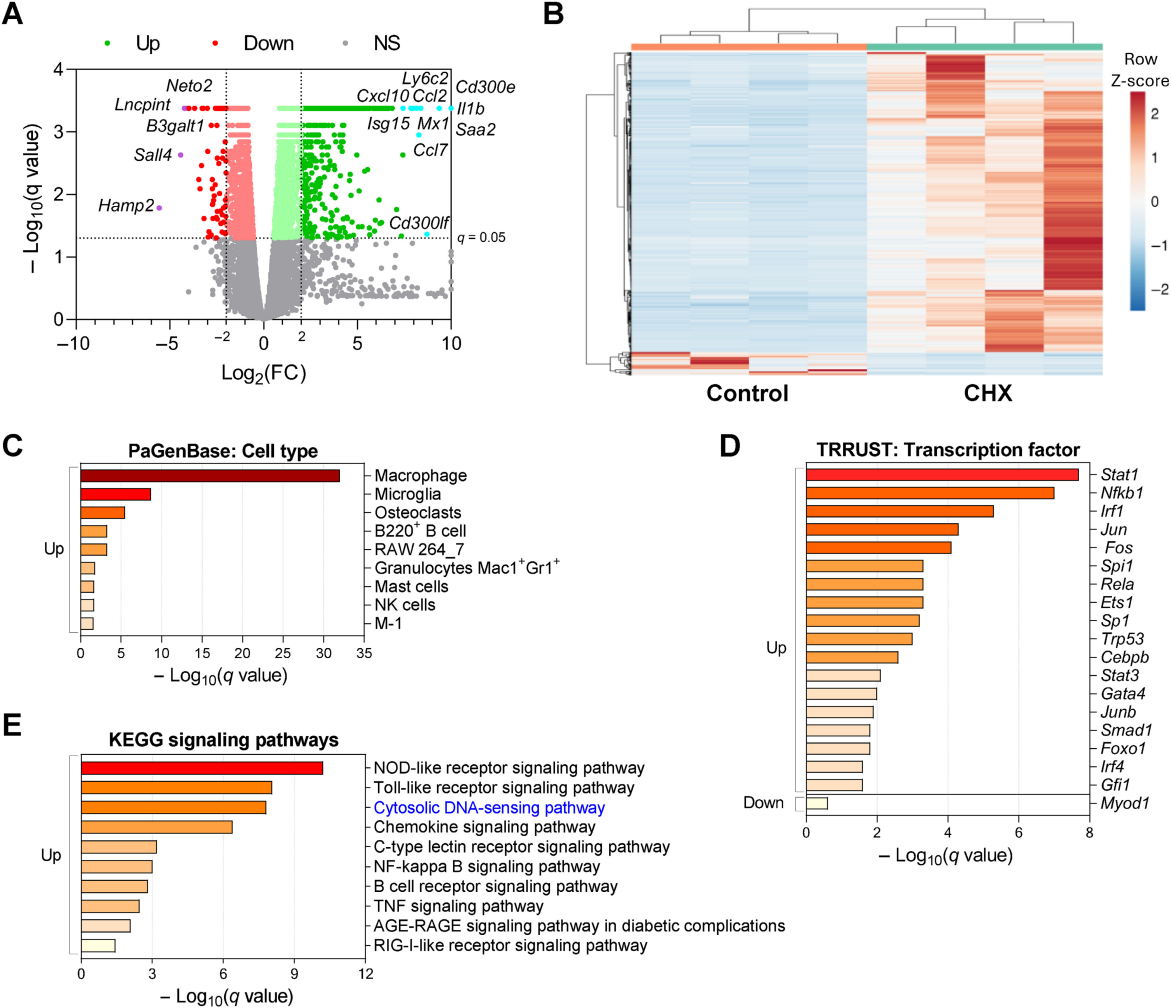
Transcriptomic and functional enrichment analysis in the peritoneum of mice exposed to CHX for 10 days. Sequencing analysis was performed starting from total RNA from parietal peritoneal tissue of male C57BL6/J mice that were i.p. injected with 0.1% CHX for 10 days and control mice (*n* = 4 per group). (A) The volcano plot shows 12,058 genes with satisfactory statistical analysis. DEGs in the CHX group versus the control group with a *q*‐value <0.05 are shown in green (increased) and red (decreased). The highest upregulated and downregulated DEGs are shown in cyan and purple, respectively. (B) Heatmap representing row *Z*‐score and the hierarchical clustering of the 1,050 DEGs with *q*‐value <0.05 and |Log_2_(FC)| ≥ 2. The heatmap was generated using the ClustVis web tool. (C–E) Functional enrichment analysis of (C) cell type, (D) transcription factors, and (E) signaling pathways in upregulated DEGs according to Pattern Gene Database (PaGenBase), Transcriptional Regulatory Relationships Unraveled by Sentence‐based Text mining (TRRUST) database, and KEGG databases, respectively. Up: Upregulated DEGs. Down: downregulated DEGs. NS: Nonsignificant.

Chlorhexidine gluconate (CHX) is a widely used agent for experimentally inducing peritoneal fibrosis in murine models, providing a useful tool for studying the pathophysiological mechanisms of PD‐associated peritoneal injury [33]. Daily i.p. injections of CHX in mice induced peritoneal inflammation as early as 3 days after starting CHX administration, peaking at 10 days (supplementary material, Figure S1A). At 10 days, parietal PM thickening with edema, angiogenesis, and infiltrating macrophages were observed, as previously reported [34], and were associated with overexpression of genes related to MMT and fibrosis. Following longer exposure to CHX, fibrosis was markedly increased, as observed here after 30 days (supplementary material, Figure S1B,C), whereas cocoon formation, a key characteristic of encapsulating peritoneal sclerosis, was described after 60 days [34]. Therefore, to identify new mediators involved in the onset of peritoneal damage, we performed an RNA‐seq study in the 10‐day CHX exposure model. From a total of 12,058 detected genes (Figure 1A), 1,050 were DEGs, with 974 upregulated and 76 downregulated (|Log_2_(FC)| ≥ 2 and *q*‐value <0.05) (Figure 1A,B; supplementary material, Table S4). The most significantly upregulated genes included proinflammatory chemokines (*Ccl2*, *Ccl7*), cytokines (*Il1b*), and ISGs (*Cxcl10*, *Isg15*) (Figure 1A; supplementary material, Table S4). To explore the potential pathophysiological associations of the transcriptomic changes, particularly the upregulated DEGs, functional enrichment analyses were conducted using Metascape‐linked bioinformatics web tools. These included Pattern Gene Database (PaGenBase) and Transcriptional Regulatory Relationships Unraveled by Sentence‐based Text mining (TRRUST) database (https://metascape.org/; accessed on 27 August 2024). This analysis found that immune cell terms were among the most represented cell types, with macrophages being the predominant cell type (Figure 1C) and NF‐κB, STAT1, and IRF1 being the main transcription factors potentially involved in the regulation of DEGs (Figure 1D). Functional analysis based on the Kyoto Encyclopedia of Genes and Genomes (KEGG) database [35] revealed classical innate immune‐related signaling pathways as the most enriched terms, among them the cytosolic DNA sensing pathway (Figure 1E). In this regard, several preclinical studies have shown that blockade of components of some of these proinflammatory pathways, such as NOD‐like receptors (NLRs), toll‐like receptors (TLRs), NF‐κB, and TNF, ameliorated peritoneal damage, [9, 36, 37, 38]. However, to date, no data about targeting cytosolic DNA‐sensing pathway have been described. In our RNA‐seq study, most components of this pathway were upregulated in the injured peritoneum of mice (Table 1; supplementary material, Figure S2). Among these, we focused further studies on the nucleotide sensor/receptor STING.

**Table 1:** Upregulated genes from cytosolic DNA‐sensing pathway in peritoneum of CHX‐treated mice compared to controls.

Classification	Gene name	Log_2_ (FC)	*q*‐value
Nucleic acid sensors	*Cgas*	4.29	0.0004
	*Sting1*	2.60	0.0004
	*Zcchc3*	2.64	0.0004
	*Ifi204 (Ifi16)*	4.96	0.0004
	*Rigi*	2.41	0.0032
	*Ddx41*	0.86	0.0026
	*Aim2*	3.79	0.0040
	*Zbp1*	6.44	0.0004
Nucleic acid‐degrading enzymes	*Adar1*	3.58	0.0004
	*Samhd1*	3.36	0.0004
	*Trex1*	1.82	0.0004
	*Dnase2a*	1.95	0.0004
DNA‐directed RNA polymerases	*Polr1c*	0.77	0.0103
	*Polr2k*	0.71	0.0114
NF‐κB pathway	*Rela*	0.97	0.0011
	*Nfkb2*	1.26	0.0004
	*Nfkbia*	1.64	0.0004
	*Nfkbie*	4.05	0.0004
	*Ikbke*	4.00	0.0004
IFN pathway	*Ifnar1*	1.51	0.0004
	*Ifnar2*	2.57	0.0004
	*Irf7*	6.12	0.0004
Cell death pathway	*Mefv (Pyrin)*	4.29	0.0004
	*Ripk3*	4.36	0.0004
	*Fadd*	1.67	0.0004
	*Pycard (Asc)*	4.73	0.0004
	*Casp1*	4.16	0.0004
	*Casp3*	1.47	0.0488
	*Casp7*	1.94	0.0004
	*Casp8*	2.40	0.0004
	*Gsdmd*	3.21	0.0004
Proinflammatory cytokines and chemokines	*Ccl4*	+	0.0004
	*Il1b*	+	0.0004
	*Cxcl10*	7.86	0.0004
	*Ccl5*	6.25	0.0004
	*Il18*	3.29	0.0008

*Note*: The table shows all the upregulated DEGs with a *q*‐value <0.05 identified in the transcriptomic analysis that belong to the cytosolic DNA‐sensing pathway (KEGG: mmu04623 https://www.kegg.jp/pathway/mmu04623).

FC, fold‐change; +, genes found expressed in peritoneum of CHX mice but with no expression detected in control mice (presence/absence genes).

### STING and other components of the cytosolic DNA‐sensing pathway are increased in the injured peritoneum of mice

**Figure 2:**
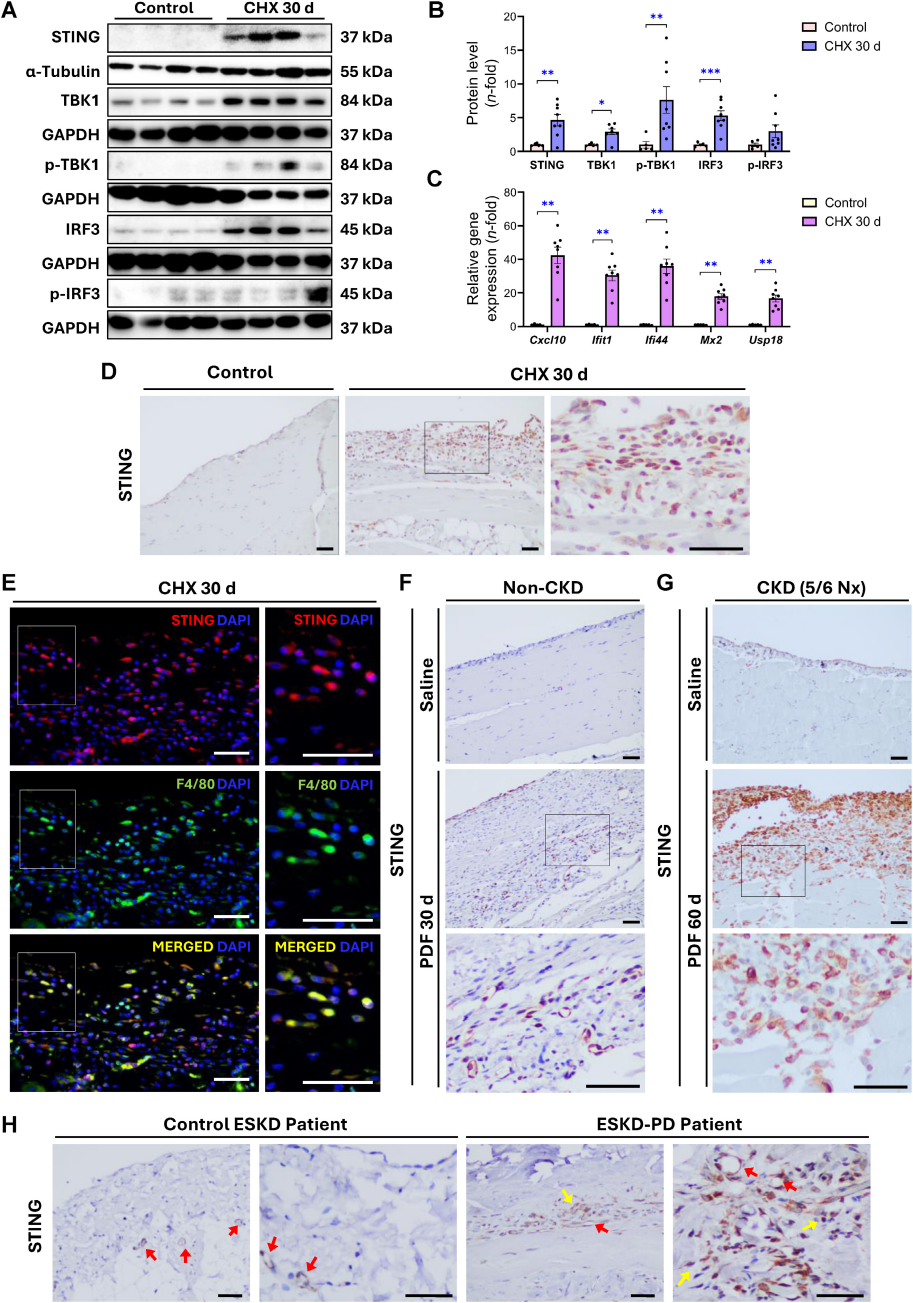
Expression and localization of STING and its downstream signaling mediators in injured peritoneal tissue from mice and patients. (A–E) Male C57BL/6J mice were daily i.p. injected with 0.1% CHX for 30 days. (A and B) Protein levels of STING, total and phosphorylated TBK1 (p‐TBK1), and total and phosphorylated IRF3 (p‐IRF3) in the peritoneum of 30‐day‐CHX‐exposed mice. Protein levels were assessed by western blotting from total proteins of parietal peritoneal tissue, using α‐tubulin or GAPDH as loading control. (C) Relative expression levels of ISGs were analyzed by RT‐qPCR from total RNA of parietal peritoneal tissue, using *Gapdh* as a housekeeping gene. Protein and gene results are expressed as *n*‐fold compared to control and represented as the mean ± SEM of five to eight animals per group. *p < 0.05, **p < 0.01, and ***p < 0.001. (D) Immunohistochemistry microscopy images showing STING^+^ cells within infiltrated and thickened areas of peritoneal membrane. (E) Immunofluorescence staining of STING (red) and F4/80 (green) shows presence of double‐labeled F4/80^+^STING^+^ (merged) macrophages in peritoneum of 30‐day‐CHX‐exposed mice. (F and G) Immunohistochemical staining of STING sections of parietal peritoneal tissue from 30‐day‐PDF‐exposed mice (F) and 60‐day‐PDF‐exposed 5/6 nephrectomized mice (G). Microscopy images in panels (D–G) correspond to a representative animal from each group. Higher‐magnification images of squared areas are presented alongside original micrographs. Nx: nephrectomy. (H) Immunohistochemical staining of STING in peritoneal biopsies from a control ESKD and an ESKD‐PD patient (Patient C1 and Patient PD1, respectively; see supplementary material, Table S1). Red arrows indicate STING^+^ stain in endothelium. Yellow arrows indicate STING^+^ stain in submesothelial zone. Scale bars, 50 μm for all micrographs.

To validate the RNA‐seq study and to confirm the involvement of the cytosolic DNA‐sensing pathway in peritoneal damage, we evaluated the expression of STING and other players of this pathway in the CHX‐induced peritoneal damage mouse model. Both gene and protein levels of STING increased in the diseased peritoneum of mice (after 3, 10, or 30 days of CHX exposure) (Figure 2A,B; supplementary material, Figure S3A,B). Consistent with this finding, the expression levels of other cytosolic DNA sensors also increased after 10 days of CHX exposure (supplementary material, Figure S3C), suggesting the presence of cytosolic DNA and the activation of the DNA‐sensing pathways. Functional STING activation in response to peritoneal damage was confirmed by the increment of the downstream machinery, including TBK1 and IRF3 (total and phosphorylated) protein levels (Figure 2A,B), as well as increased expression of ISGs after 10 days and 30 days of CHX administration (Figure 2C; supplementary material, Figure S3D). Together with IFN signaling activation, the levels of phosphorylated NF‐κB‐inhibitor‐α (p‐IκBα) were increased in the damaged peritoneum, indicating the activation of the NF‐κB pathway (supplementary material, Figure S3E). In summary, these data indicate the activation of STING signaling in response to experimental peritoneal damage.

### STING‐expressing cells are abundantly found in the chronically injured peritoneum of CHX‐ and PDF‐exposed mice and PD patients

In the peritoneal tissue of 30‐day‐CHX‐treated mice, STING^+^ cells were found in the submesothelial zone in areas of both PM thickening and inflammatory cell infiltration (Figure 2D). Furthermore, consistent with macrophages being the most enriched cell subset in the transcriptomic analysis (Figure 1C), F4/80^+^ macrophages expressing STING were abundantly found in the damaged peritoneum of mice (Figure 2E). We next further evaluated whether STING was modulated by long‐term exposure to conventional PDFs. To this end, two preclinical models of chronic peritoneal exposure to PDF, mimicking PD therapy in normal mice (non‐CKD) and mice with 5/6 nephrectomy (CKD), were established as previously described [30]. STING^+^ cells were abundant in the peritoneum of PDF‐exposed mice, in either the absence or presence of kidney insufficiency, whereas these cells were scarce in healthy peritoneum (Figure 2F,G). To explore the clinical relevance of these findings, we examined the presence of STING^+^ cells in peritoneal biopsies from ESKD patients undergoing PD treatment. Strong positive staining for STING was found in cells of the submesothelial zone in biopsies of ESKD‐PD patients, but not in control ESKD samples (Figure 2H; supplementary material, Figure S4). Interestingly, STING^+^ staining was found in the peritoneal endothelium of both control ESKD and ESKD‐PD cases. Accordingly, STING‐expressing endothelial cells were identified in the peritoneum of PDF‐injured mice, as evidenced by double immunofluorescence staining of STING and CD31 (supplementary material, Figure S5). Therefore, STING was upregulated in the damaged peritoneum of both PDF‐exposed mice and CKD patients undergoing PD, supporting its potential role in promoting peritoneal injury during PD therapy.

### STING deficiency ameliorates early CHX‐induced peritoneal inflammation

**Figure 3:**
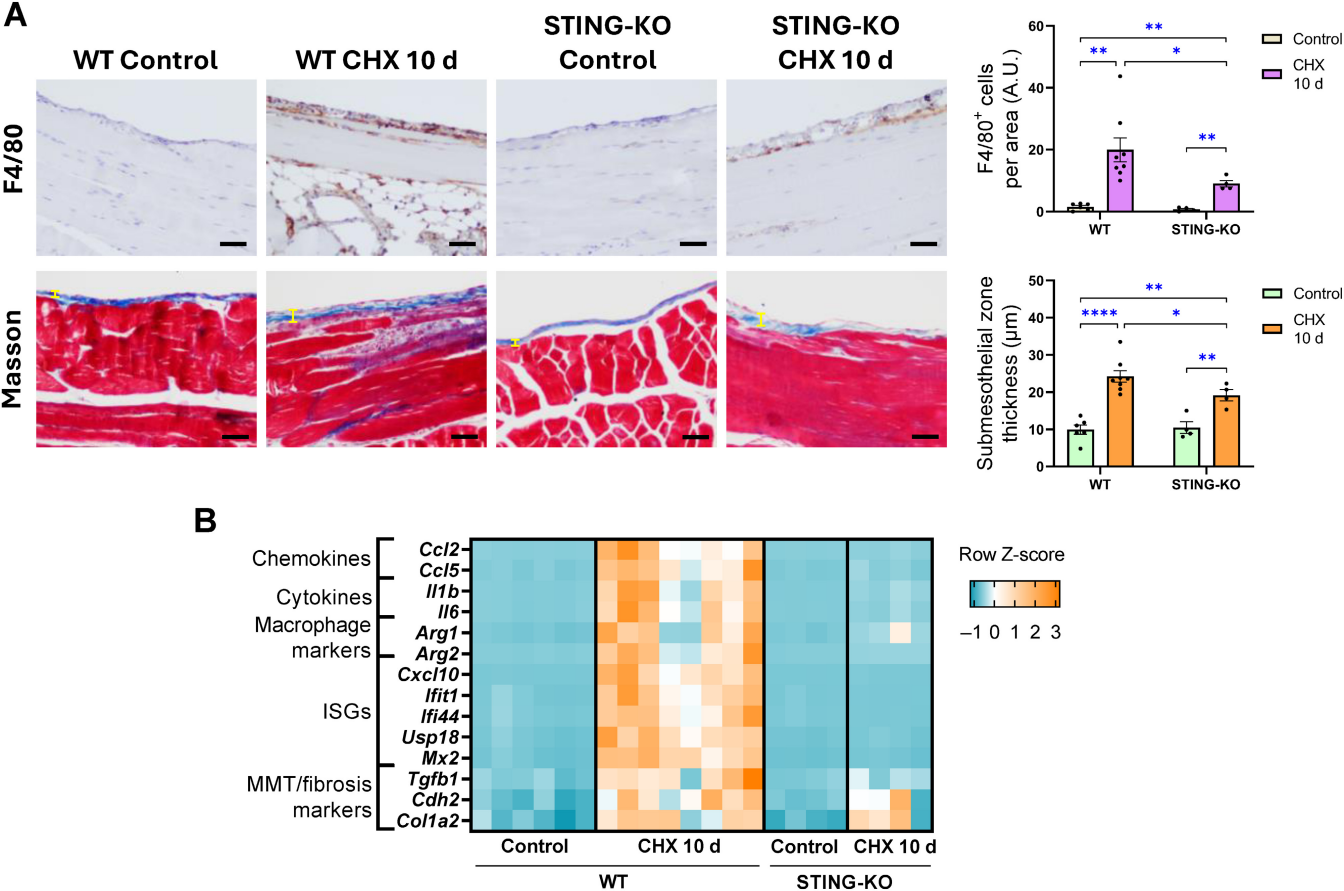
Peritoneal damage in WT and STING‐KO mice exposed to CHX for 10 days. Male C57BL/6J WT and STING‐deficient (STING‐KO) mice were i.p. injected with 0.1% CHX for 10 days. (A) Peritoneal macrophage infiltrates and peritoneal membrane thickness in CHX‐exposed mice. Upper panel: F4/80 immunohistochemistry images from a representative animal from each group (left) and the corresponding quantification of F4/80^+^ cells (right). Bottom panel: representative Masson's trichrome stained images (left) and corresponding quantification of submesothelial zone thickness (right). Yellow lines indicate width measured. Scale bar, 50 μm. Results are represented as mean ± SEM of four to eight animals per group. (B) Heatmap representing row *Z*‐score of relative expression levels of inflammatory and MMT/fibrosis markers. Relative gene expression was analyzed by RT‐qPCR from total RNA of parietal peritoneal tissue, using *Gapdh* as a housekeeping gene. *p < 0.05, **p < 0.01, and ****p < 0.0001. A.U.: arbitrary unit

To unravel the role of STING in the development of peritoneal damage, the CHX‐induced peritoneal injury model was replicated in STING‐deficient (STING‐KO) mice. After 10‐day‐CHX exposure, the absence of STING significantly reduced CHX‐induced macrophage infiltration into the peritoneal tissue (Figure 3A) and inhibited gene upregulation of several inflammatory markers (Figure 3B). Additionally, CHX‐exposed STING‐KO mice exhibited a slight, but significant, decrease in PM thickness compared to WT mice (Figure 3A), together with inhibited CHX‐induced overexpression of *Tgfb1*, but no other MMT/fibrosis markers (Figure 3B). Therefore, these data clearly demonstrate that STING participates in the inflammatory response in the injured peritoneum.

### STING deficiency attenuates chronic CHX‐induced peritoneal inflammation and fibrosis

**Figure 4:**
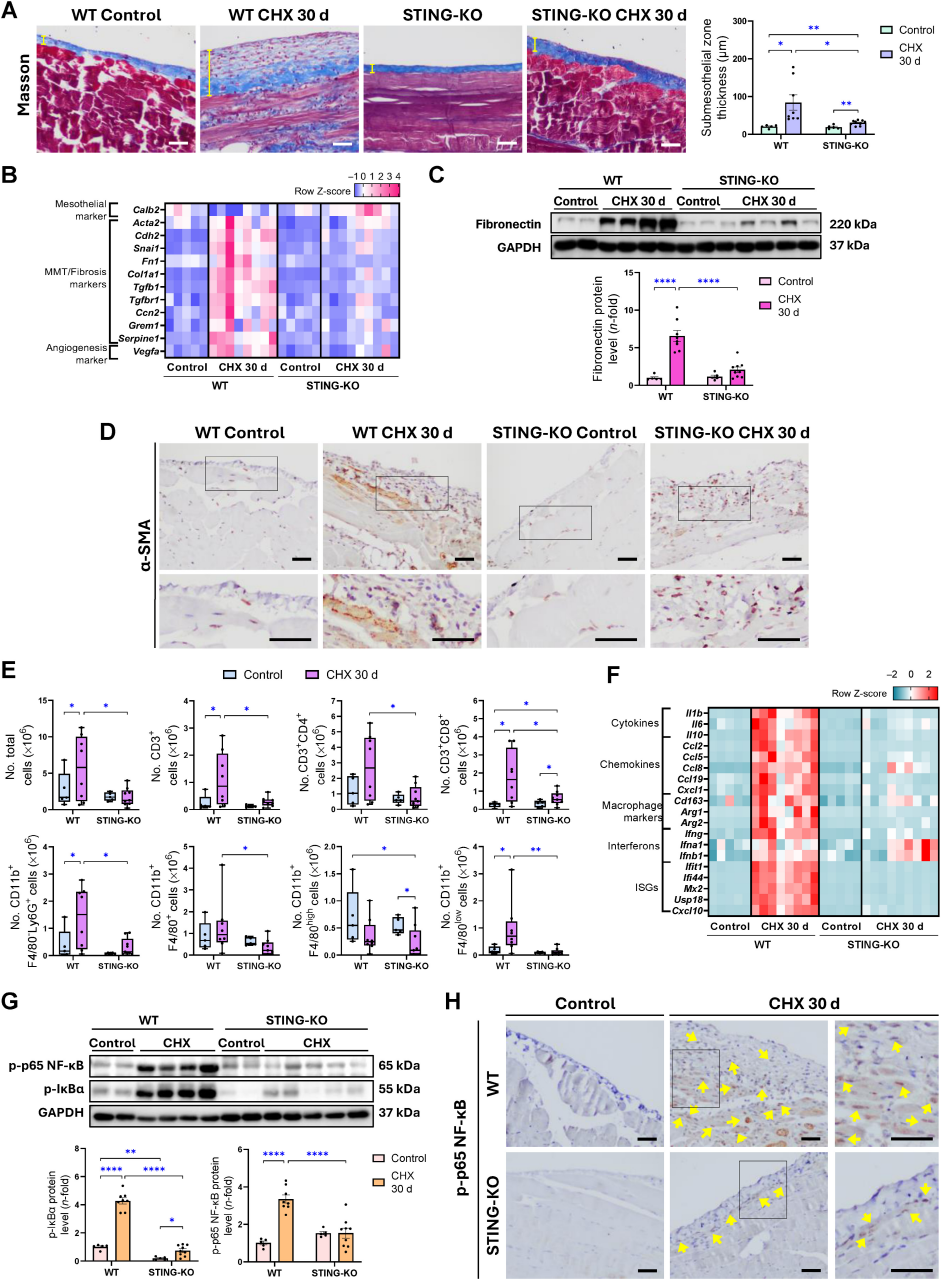
Peritoneal membrane thickening, fibrosis, and inflammation in WT and STING‐KO mice exposed to CHX for 30 days. Male C57BL/6J WT and STING‐deficient (STING‐KO) mice were i.p. injected with 0.1% CHX for 30 days. (A) Masson's trichrome staining in parietal peritoneal tissue. Yellow lines in Masson's trichrome images indicate width measured. (B) Heatmap representing row *Z*‐score of relative expression levels of mesothelial marker calretinin (*Calb2*) and several MMT/fibrosis and angiogenesis markers. (C) Fibronectin protein levels in parietal peritoneum of mice (D) α‐SMA immunostaining of parietal peritoneal tissue from mice. (E) Flow cytometry analysis in peritoneal lavages from mice to identify cells present in peritoneal cavity. Peritoneal cells were labeled with specific antibodies tagged with different fluorophores to identify CD3^+^, CD4^+^, CD8^+^, CD11b^+^, Ly6G^+^, and F4/80^+^ cells. Cytometry results are represented in scattered box plots with min‐to‐max whiskers, and the median and quartiles are shown. (F) Heatmap representing row *Z*‐score of relative expression levels of inflammatory markers. (G) Protein levels of phosphorylated p65 subunit of NF‐κB (p‐p65 NF‐κB) and phosphorylated IκBα (p‐IκBα) in parietal peritoneum of mice. (H) Immunohistochemical detection of phosphorylated (p)‐p65 NF‐κB protein in parietal peritoneal tissue sections. Yellow arrows indicate p‐p65^+^ nuclei. Microscopy images in panels (A), (D), and (H) correspond to a representative animal from each group. Higher‐magnification images of the squared areas in panels (D) and (H) are presented alongside the original micrographs. Scale bars, 50 μm for all micrographs. Relative gene expression was analyzed by RT‐qPCR from total RNA of parietal peritoneal tissue, using *Gapdh* as a housekeeping gene. Protein levels were assessed by western blotting from total proteins of parietal peritoneal tissue, using GAPDH as loading control. Western blotting results are expressed as fold change (*n*‐fold) relative to control group. Results are represented as mean ± SEM of five to nine animals per group. *p < 0.05, **p < 0.01, ****p < 0.0001.

To further evaluate the role of STING in peritoneal fibrosis and persistent inflammation, studies were performed in STING‐KO mice chronically exposed to CHX for 30 days. STING deficiency significantly reduced CHX‐induced PM thickening compared to WT mice (Figure 4A). Additionally, gene expression of the MC marker calbindin 2 (*Calb2* gene) was decreased after CHX exposure in WT mice but not in STING‐deficient mice (Figure 4B), suggesting that STING may play a role in MC loss. Moreover, STING deficiency inhibited CHX‐induced peritoneal gene overexpression of MMT/fibrosis markers (Figure 4B), in association with decreased fibronectin protein levels (Figure 4C) and reduced peritoneal staining of the myofibroblastic cell marker, α‐smooth muscle actin (α‐SMA) (Figure 4D). These findings support the potential role of STING in promoting MMT and fibrosis in the peritoneum. Peritoneal inflammation persisted after 30 days of CHX exposure in WT mice (Figure 4E–H). However, while flow cytometry analysis of peritoneal lavages from WT mice showed a significant increase in the number of immune cells recruited into the peritoneal cavity, a notable reduction was observed in STING‐KO mice (Figure 4E). T cells, macrophages, and neutrophils were among the cell subsets that displayed lowered peritoneal cavity recruitment and tissue infiltration in STING‐KO mice (Figure 4E; supplementary material, Figure S6). Additionally, peritoneal levels of proinflammatory genes were upregulated in CHX‐exposed WT mice, but no alterations were observed in most of these genes in CHX‐exposed STING‐KO mice compared to control mice (Figure 4F). Among the inflammatory pathways induced in CHX mice, NF‐κB was one of the most upregulated (Figure 1E). This activation of the NF‐κB pathway was prevented by STING deficiency, as indicated by the decrease in both p‐IκBα and p‐p65 NF‐κB levels (Figure 4G,H). Moreover, CHX‐induced gene upregulation of proangiogenic factors, such as *Vegfa* and *Cxcl1*, was also decreased in the absence of STING (Figure 4B,F). Together, these findings demonstrate that STING participates in peritoneal fibrosis, angiogenesis, and inflammation induced by chronic and repeated exposure to CHX.

### Pharmacological inhibition of STING ameliorates CHX‐induced peritoneal damage

**Figure 5:**
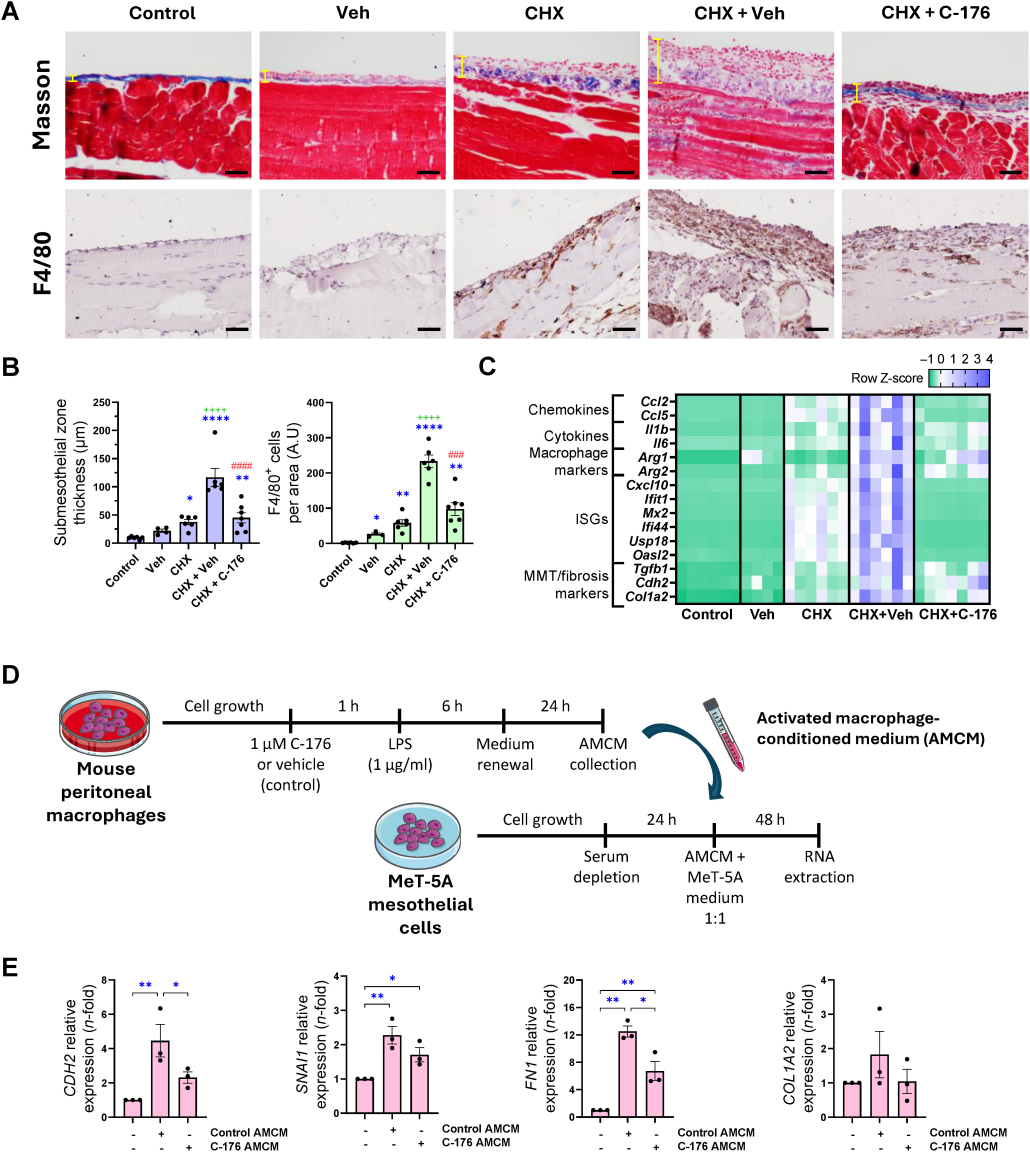
*In vivo* and *in vitro* effects of STING pharmacological inhibition. (A–C) Male C57BL/6J WT mice were i.p. injected with 0.1% CHX together with pharmacological STING inhibitor C‐176 or its vehicle (Veh, corn oil) for 10 days. Untreated and vehicle‐treated mice were used as controls. (A) Peritoneal membrane thickness (upper panel) and immunohistochemical staining of F4/80 (bottom panel). Microscopy images correspond to a representative animal from each group. Yellow lines indicate thickness measured in Masson's trichrome images. Scale bar, 50 μm. (B) Graphs showing corresponding quantification of submesothelial zone thickness (left) and F4/80^+^ cells (right). Results are represented as mean ± SEM of four to seven animals per group. *p < 0.05, **p < 0.01, ****p < 0.0001 compared to control group. ++++*p* < 0.0001 compared to CHX group. ###*p* < 0.001, ####*p* < 0.0001 compared to CHX + Veh group. (C) Heatmap representing row *Z*‐scores of relative expression levels of inflammatory and MMT/fibrosis markers. Relative gene expression was analyzed by RT‐qPCR from total RNA of parietal peritoneal tissue using *Gapdh* as housekeeping gene. (D) Schedule of *in vitro* study design for assessment of macrophage‐mesothelial cell interaction. Created using elements from Servier Medical Art (Servier; https://smart.servier.com/). (E) Relative gene expression of MMT and fibrosis markers in MeT‐5A cells incubated for 48 h with pooled conditioned medium from LPS‐activated macrophages (AMCM) with or without C‐176 treatment. Relative gene expression levels were analyzed by RT‐qPCR from total RNA using *GAPDH* as housekeeping gene, expressed as fold‐change (*n*‐fold) relative to control condition (first column) and represented as mean ± SEM of three to five independent experiments. *p < 0.05, **p < 0.01, ***p < 0.001, ****p < 0.0001. A.U.: arbitrary unit.

To test the *in vivo* potential protective effect of STING pharmacological inhibition in peritoneal damage, we developed a 10‐day CHX exposure model in WT mice together with daily i.p. administration of the covalent STING inhibitor C‐176 (750 nmol/mouse, dissolved in corn oil as the vehicle). Vehicle administration significantly enhanced CHX‐induced peritoneal damage compared with CHX‐only exposure (Figure 5A–C). Despite the harmful effect of vehicle, C‐176 treatment was able to significantly attenuate CHX‐induced PM thickening and macrophage infiltration in comparison to CHX + vehicle group (Figure 5A,B). Moreover, C‐176 treatment restored the gene expression levels of a panel of inflammatory genes and attenuated the induction of macrophage and MMT/fibrosis markers (Figure 5C). Taken together, these results demonstrate that pharmacological inhibition of STING improves the peritoneal injury caused by CHX in mice.

### Pharmacological targeting of STING inhibits macrophage‐mediated MMT *in vitro*

MCs can undergo MMT, acquiring a myofibroblast phenotype that contributes to extracellular matrix accumulation leading to fibrosis [39]. To evaluate the direct impact of STING on the MMT process, MeT‐5A MCs were pretreated with the STING inhibitor H‐151 and then stimulated with TGF‐β + IL‐1β for 24 h. No significant changes were observed in the gene expression of MMT and fibrosis markers following H‐151 treatment compared to TGF‐β + IL‐1β stimulation (supplementary material, Figure S7A). Despite this, the STING pathway remained responsive in MCs when challenged with TNF‐α for 24 h, as evidenced by induced gene expression of chemokines and ISGs, which were dampened by H‐151 (supplementary material, Figure S7B). Therefore, these results suggest that STING does not directly regulate MMT in MCs but contributes to the maintenance of the inflammatory response. Since STING^+^ macrophages were detected in CHX‐injured peritoneum (Figure 2E), we examined whether STING inhibition in macrophages could influence MMT in cultured MCs. To this aim, mouse peritoneal macrophages were collected, cultured, and then activated with LPS, with or without C‐176 pretreatment. We then used macrophage supernatants as activated macrophage‐conditioned media (AMCM) to stimulate MeT‐5A MCs and evaluate MMT (Figure 5D). Control AMCM (supernatant from macrophages not pretreated with C‐176) induced a significant increase in the gene expression of MMT/fibrosis markers, which was further prevented in MCs treated with AMCM from C‐176‐pretreated macrophages (C‐176 AMCM) (Figure 5E). These results suggest that STING activation in macrophages modulates responses in MCs and it may be involved in peritoneal MMT and fibrosis.

### STING deficiency attenuates postsurgical peritoneal adhesions

**Figure 6:**
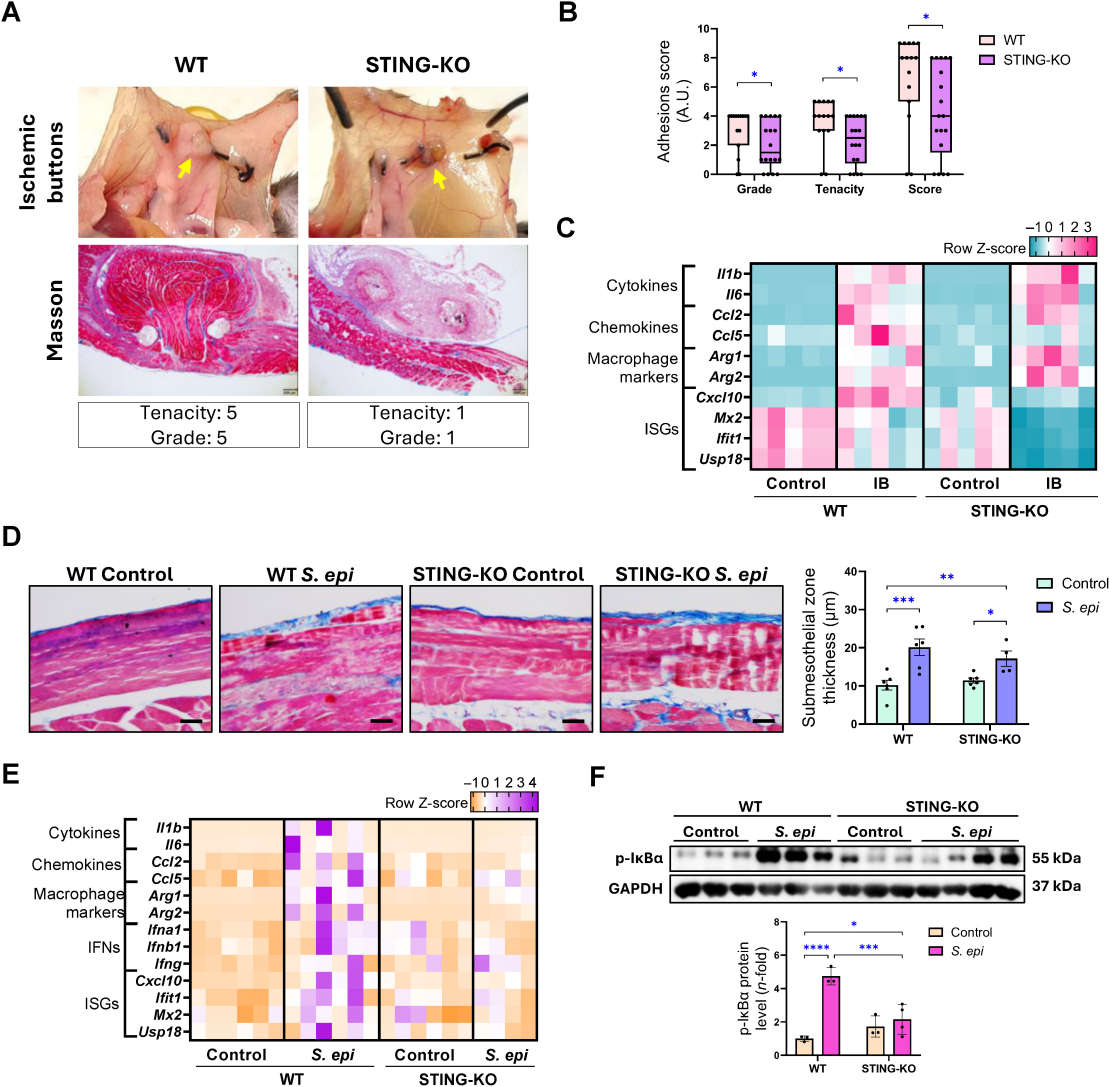
Effect of STING deficiency on peritoneal adhesion formation and peritonitis development in mice. (A–C) For peritoneal adhesion model, three IBs were surgically made in peritoneum of male C57BL/6J WT and STING‐deficient (STING‐KO) mice. Adhesion formation on IBs was assessed 5 days after surgery. (A) Macroscopic and microscopic analyses of adhesions formed on IBs surgically made in mice. Yellow arrows in top panel indicate IB to which correspond lower Masson's trichrome micrographs (scale bar, 200 μm). Microscopy images correspond to a representative animal from each group. (B) Peritoneal adhesion grade, tenacity, and total score values are represented in scattered box plots with min‐to‐max whiskers, and the median and quartiles are shown for each experimental group (*n* = 5–6 mice per group, 3 IBs/mouse). (C) Heatmap representing row *Z*‐scores of relative expression levels of inflammatory markers. (D–F) For peritonitis model, female C57BL/6J WT and STING‐deficient (STING‐KO) mice were injected with a single dose of live *S. epidermidis* bacteria (*S. epi*, 5 × 10^8^ cfu/mouse) and evaluated 72 h after injection. (D) Peritoneal membrane thickness assessment. The figure shows Masson's trichrome‐stained parietal peritoneal tissue sections of representative mice from each group (left; scale bar, 50 μm) and the corresponding quantification of submesothelial zone thickness (right). Yellow lines indicate width measured. (E) Heatmap representing row *Z*‐scores of relative expression levels of inflammatory markers. (F) Protein levels of phosphorylated IκBα (p‐IκBα) were assessed by western blotting from total proteins of parietal peritoneum using GAPDH as loading control. Protein‐level results are expressed as fold‐change (*n*‐fold) relative to control group. Relative gene expression was analyzed by RT‐qPCR from total RNA of parietal peritoneal tissue, using *Ppia* as a housekeeping gene. Results are represented as mean ± SEM of three to six animals per group. *p < 0.05, **p < 0.01, ***p < 0.001, ****p < 0.0001. A.U.: Arbitrary unit.

The role of STING in the development of peritoneal adhesions was evaluated in a model of intra‐abdominal adhesions induced by surgically generated ischemic buttons (IBs), as previously described [40]. Abundant STING^+^ cells were found in peritoneal areas close to IBs and in the peritoneum‐organ adhesion interface in WT mice (supplementary material, Figure S8A,B). Accordingly, increased protein levels of STING, TBK1, IRF3, and p‐IκBα were observed in the IB tissue (supplementary material, Figure S8C). In STING‐deficient mice, the extent of the IB covered by the adhesion (grade), the tenancy, and the total score of the adhesions (scoring according to details provided in supplementary material, Table S2) were significantly decreased compared to WT mice (Figure 6A,B). Infiltration of macrophages and T cells was also reduced in IBs from STING‐deficient mice compared to WT mice (supplementary material, Figure S9). Moreover, gene expression levels of proinflammatory markers *Ccl2*, *Cxcl10*, and other ISGs were reduced in the IBs from STING‐KO mice compared to WT mice (Figure 6C). Together, these results show that STING deficiency ameliorates peritoneal adhesions in mice.

### STING deficiency alleviates *S. epidermidis*‐induced peritonitis

*S. epidermidis* is one of the main causes of bacterial peritonitis in PD patients [41, 42]. To evaluate the participation of STING in peritonitis, a single dose of live *S. epidermidis* (5 × 10^8^ CFU/mouse) was injected into STING‐KO and WT mice, as previously described [43, 44, 45]. Peritoneal tissue was then examined after 72 h. *S. epidermidis* administration increased parietal PM thickening with edema in both WT and STING‐KO mice (Figure 6D). STING deficiency was able to prevent *S. epidermidis*‐induced upregulation of inflammatory genes, such as chemokines, cytokines, macrophage markers, IFNs, and ISGs (Figure 6E). STING‐KO mice also showed decreased *S. epidermidis*‐induced p‐IκBα levels, suggesting reduced activation of the NF‐κB pathway compared to WT mice (Figure 6F). Therefore, STING deficiency improved peritoneal inflammation induced by *S. epidermidis* in mice.

## Discussion

In this study, we report for the first time that the STING pathway contributes to peritoneal damage across multiple preclinical models of PD‐associated injury, including exposure to chemical agents (CHX and PDF), peritoneal adhesions, and bacterial peritonitis. Both genetic deletion of STING and its pharmacological inhibition reduced peritoneal inflammation and fibrosis, underscoring the role of this pathway in initiating and propagating damage triggered by sterile and nonsterile insults that engage innate immune responses to cytoplasmic DNA accumulation (supplementary material, Figure S10). Moreover, STING expression was detected in injured peritoneal tissue from ESKD patients undergoing PD, highlighting its potential clinical relevance. Preclinical evidence also supports STING inhibition as a strategy to mitigate renal injury by targeting inflammation‐driven tissue damage [46, 47, 48, 49, 50]. Although PD is a life‐saving therapy for ESKD patients, repeated and prolonged exposure to PDFs can lead to peritoneal damage and complications, including bacterial infections and catheter adhesions, which impair ultrafiltration efficiency and may result in therapy discontinuation [51]. Based on these findings, we propose STING as a promising therapeutic target to prevent PD‐associated peritoneal deterioration.

Transcriptomic analyses of peritoneal biopsies from patients undergoing PD and nondialyzed uremic controls revealed a significant enrichment of inflammatory and immune‐related pathways [52]. Consistently, in a CHX‐induced model mimicking chronic PDF exposure, we found that the most upregulated genes were associated with inflammatory pathways, including chemokines, cytokines, and ISGs. Functional enrichment analysis further identified innate immune pathways, such as the cytosolic DNA‐sensing pathway – previously unreported in this context – as well as known mediators of PD‐associated damage, including those relying on NLRs, TLRs, NF‐κB, and TNF‐α. Preclinical studies have demonstrated that targeting these innate proinflammatory pathways can prevent peritoneal damage [9, 36, 37, 38, 53, 54, 55, 56], as we demonstrate here by targeting STING.

Activation of STING promotes the recruitment of TBK1 and IKKε, which in turn activate the canonical type I interferon response and the NF‐κB signaling pathway, respectively, ultimately contributing to tissue inflammation and injury [20, 23, 57, 58, 59]. Our transcriptomic analysis revealed that NF‐κB was a central regulator of proinflammatory gene expression programs in the peritoneal damage model. Previous studies demonstrated that pharmacological inhibition of NF‐κB using parthenolide, a well‐characterized IKKα/β inhibitor attenuated peritoneal injury by suppressing inflammatory responses [9, 60]. Remarkably, NF‐κB activation was significantly attenuated in the murine peritoneum following CHX‐induced injury in STING‐deficient mice, as well as upon pharmacological treatment with C‐176, a small‐molecule inhibitor of STING [61]. Consistently, C‐176 treatment suppressed the expression of NF‐κB–dependent cytokines, thereby reducing immune cell infiltration into the CHX‐exposed peritoneal tissue. These findings are in line with the reported anti‐inflammatory effects of the STING inhibitor H‐151 in experimental dermatitis [62], and with the protective role of STING deficiency against hyperglycemia‐induced NF‐κB activation and oxidative mitochondrial damage in endothelial cells *in vivo* [63]. Moreover, in our model of peritonitis induced by live *S. epidermidis* administration, STING deficiency resulted in reduced NF‐κB activation and downregulation of inflammatory chemokines, cytokines, and macrophage‐associated markers. These findings indicate that STING–mediated NF‐κB signaling contributes to the inflammatory response during sterile and infection‐induced peritoneal injury.

PD‐associated peritonitis remains a major clinical challenge due to its high incidence and strong association with increased cardiovascular mortality [64, 65]. To mitigate this long‐term risk, combined anti‐inflammatory and antimicrobial therapies have been proposed [66]. In this context, inhibition of the STING pathway may arise as a promising strategy, with preclinical evidence supporting its cardiovascular benefits through both genetic and pharmacological approaches [67, 68, 69, 70, 71]. Furthermore, in a murine model of bacterial peritonitis, calprotectin inhibition – a DAMP and TLR4 ligand – significantly reduced both acute and chronic systemic and vascular inflammation without compromising bacterial clearance [45]. Similarly, the CANTOS trial and related studies using IL‐1β monoclonal antibodies demonstrated that targeting chronic inflammation reduced cardiovascular risk, particularly in CKD patients, although at the cost of increased infection‐related mortality [72, 73]. These findings underscore the need for targeted anti‐inflammatory strategies, such as TLR4 or STING inhibition, especially in infection‐prone populations undergoing chronic PD.

STING is widely expressed in both resident and immune cell populations [58]. In human peritoneal biopsies, STING^+^ cells were primarily localized within the submesothelial compact zone, particularly in endothelial and infiltrating cells in fibrotic regions. Notably, STING activation in endothelial cells was observed in peritoneal biopsies from ESKD patients, regardless of PD therapy, suggesting that systemic CKD‐related factors may also contribute. As noted previously, peritoneal biopsies from patients undergoing PD and nondialyzed uremic controls present activation of inflammatory and immune‐related responses [52]. Preclinical studies further support a pathogenic role of STING in endothelial dysfunction. Endothelial‐specific STING deletion reduces lipid accumulation and improves both vascular function [74] and insulin sensitivity [75]. STING signaling also promotes endothelial senescence through IRF3 and NF‐κB [76] and contributes to angiogenesis via an independent mechanism [77]. Consistent with these findings, our preclinical studies revealed that PDF‐exposure activated STING in endothelial cells, while STING inhibition attenuated *Vegfa* and *Cxcl1* overexpression in CHX‐induced peritoneal injury, both key mediators of peritoneal angiogenesis in PD [78, 79]. Given the established role of STING in cardiovascular disease [67, 68, 69, 70, 71] and endothelial dysfunction [74, 75, 76, 77], further investigation is warranted to elucidate the biological implications of these observations.

Inflammatory cell infiltration is a hallmark of peritoneal injury [7]. In a murine model of CHX‐induced peritoneal injury, transcriptomic analysis identified macrophages as the most enriched STING‐expressing population, a finding corroborated by immunostaining showing STING^+^ infiltrating macrophages. Notably, both genetic deficiency and pharmacological inhibition of STING significantly reduced macrophage infiltration in this model. Consistently, STING inhibition also attenuated macrophage recruitment and the expression of proinflammatory genes in models of bacterial peritonitis and peritoneal adhesions. The presence of STING^+^ macrophages in regions of peritoneal thickening and fibrosis suggests a potential role for STING in macrophage‐driven fibrogenesis/MMT. Recent evidence has linked STING activation to macrophage immunometabolic reprogramming [80]. Supporting this, our *in vitro* experiments using conditioned media from LPS‐activated macrophages on MCs demonstrated that STING pharmacological inhibition in macrophages reduced MMT induction. Collectively, these findings underscore a functional crosstalk between immune cells and the peritoneal stroma, providing evidence that STING activation in macrophages contributes to peritoneal fibrosis and MMT *in vivo*.

Peritoneal fibrosis is an adverse outcome of chronic exposure to PDFs, characterized by progressive thickening of the submesothelial compact zone and membrane dysfunction [81]. A rare but severe complication of long‐term PD is encapsulating peritoneal sclerosis (EPS), marked by fibrosis and adhesions of the peritoneum to the small bowel loops, resulting in intestinal obstruction and potentially fatal consequences [82]. Surgical interventions, including PD catheter insertion, may also contribute to peritoneal adhesions formation [12, 83]. Additionally, intra‐abdominal adhesions are post‐inflammatory complications that can compromise PD efficacy [11, 12]. Our experimental data in STING‐deficient mice revealed reduced fibrosis following prolonged CHX exposure and fewer peritoneal adhesions after surgery, indicating that STING inhibition modulates fibrosis‐related responses. STING inhibition has been shown to attenuate fibrosis in human and mouse hypertrophic cardiomyopathy [84] as well as in experimental models of kidney, lung, and liver fibrosis [85, 86]. Conversely, STING deficiency unexpectedly exacerbated lung fibrosis in a type I IFN‐independent manner [87]. However, a protective role for STING against fibrosis has been reported in chronic pancreatitis [88], underscoring the context‐dependent nature of STING signaling in fibrotic processes. Biochemical mediators and biomechanical forces converge to activate TGF‐β1, initiating signaling cascades that drive MMT, a central mechanism in peritoneal fibrosis [89, 90, 91]. A key finding of our study is that both genetic and pharmacological STING targeting reduces CHX‐induced peritoneal fibrosis by downregulating fibrotic mediators, such as TGF‐β1, and restoring mesothelial marker expression, thereby preventing MMT. We previously characterized the transcriptomic reprogramming of MC toward a myofibroblast‐like phenotype, defining a panel of pro‐MMT markers [92]. Our *in vitro* data demonstrate that STING does not directly induce MMT in MCs. However, STING contributes to the inflammatory response in these cells, as evidenced by the suppression of TNFα‐induced proinflammatory gene expression following treatment with the STING inhibitor H‐151. Although STING may modulate fibrotic responses in a context‐dependent manner, our findings support a key role for STING signaling in sustaining chronic inflammation. This persistent proinflammatory loop contributes to MMT and fibrosis. Therefore, targeting STING emerges as a promising therapeutic strategy to counteract pathological remodeling and preserve peritoneal membrane function during PD.

Although our study is based on murine models, which may not fully recapitulate human pathophysiology, it provides valuable insights by identifying STING as a key regulator of peritoneal injury progression and a potential therapeutic target for preventing peritoneal damage. Nonetheless, pharmacological strategies targeting STING remain at the preclinical stage. To date, clinical trials addressing STING modulation have focused exclusively on the use of STING agonists in oncology (NCT04020185 https://clinicaltrials.gov/study/NCT04020185; NCT03172936 https://www.clinicaltrials.gov/study/NCT03172936; NCT04609579 https://clinicaltrials.gov/study/NCT04609579), with the use of inhibitors pending development. Our findings demonstrating the presence of STING‐activated cells in peritoneal biopsies from PD patients underscore the translational relevance of preclinical models to human disease. Nonetheless, future studies incorporating non‐CKD control tissues are essential to more precisely delineate disease‐specific alterations. In summary, our findings highlight STING as a novel mediator of immune and inflammatory responses associated with PD‐related peritoneal injury, which may result in therapy discontinuation, a major clinical concern in patients undergoing PD. Future research is warranted to develop and evaluate STING inhibitors for clinical use aimed at preserving PM function and ultrafiltration capacity in PD patients, alongside potential benefits in managing other CKD‐related complications.

## Author contributions statement

VM, GTG‐M, A‐CR, ML‐C, AMR and MR‐O conceived and designed the work. VM, JG‐J, GTG‐M, RR, PS, LT‐S and SR‐M performed the experiments and obtained the data. VM, JG‐J and PS performed animal models. VM, GTG‐M, PS, RR, JAJ‐H, AMR and MR‐O analyzed and interpreted the results. VM, AMR and MR‐O drafted the manuscript. VM, AMR, AO and MR‐O edited and revised the manuscript. All authors approved the final version of the manuscript.

## Supporting information


Supplementary materials and methods
**Figure S1.** Time course of peritoneal damage induced by CHX in mice
**Figure S2.** Upregulated genes of cytosolic DNA‐sensing pathway in peritoneum of 10‐day‐CHX‐exposed mice
**Figure S3.** Expression of STING and other cytosolic DNA sensors in peritoneum of CHX‐exposed mice
**Figure S4.** STING^+^ staining in peritoneal biopsies from PD patients
**Figure S5.** STING^+^ staining in peritoneal endothelium PDF‐exposed mice
**Figure S6.** Inflammatory cell infiltration into peritoneum of WT and STING‐KO mice exposed to CHX for 30 days
**Figure S7.** Pharmacological inhibition of STING in cultured mesothelial cells
**Figure S8.** Expression of STING and its downstream signaling mediators in postsurgical peritoneal adhesions
**Figure S9.** Immune cell infiltration in peritoneal adhesions of WT and STING‐KO mice
**Figure S10.** STING‐mediated cytosolic DNA‐sensing pathway as new pathogenic mechanism in peritoneal damage
**Table S1.** Demographic and clinical characteristics of control and PD patients
**Table S2.** Postsurgical adhesion mouse scoring scheme
**Table S3.** Predesigned assays used for qPCR
**Table S4.** DEGs on peritoneum from CHX‐treated mice versus control mice

## Data Availability

The RNA‐seq data sets analyzed in this study are available from the Gene Expression Omnibus repository under the following accession number: GSE282440 https://www.ncbi.nlm.nih.gov/geo/query/acc.cgi?acc=GSE282440. Data are reported following the MINSEQE guidelines (https://www.fged.org/projects/minseqe/). All data are available from the corresponding author upon reasonable request.
