# Supplementary material for: STING inhibition alleviates experimental peritoneal damage: potential therapeutic relevance for peritoneal dialysis
Source: J Pathol. 2025 Aug 14;267(2):196–212. doi: 10.1002/path.6462 (PMC12438029; doi:10.1002/path.6462)
Supplement: Supplementary file 1 — Supplementary materials and methods Figure S1. Time course of peritoneal damage induced by CHX in mice Figure S2. Upregulated genes of cytosolic DNA‐sensing pathway in peritoneum of 10‐day‐CHX‐exposed mice Figure S3. Expression of STING and other cytosolic DNA sensors in peritoneum of CHX‐exposed mice Figure S4. STING+ staining in peritoneal biopsies from PD patients Figure S5. STING+ staining in peritoneal endothelium PDF‐exposed mice Figure S6. Inflammatory cell infiltration into peritoneum of WT and STING‐KO mice exposed to CHX for 30 days Figure S7. Pharmacological inhibition of STING in cultured mesothelial cells Figure S8. Expression of STING and its downstream signaling mediators in postsurgical peritoneal adhesions Figure S9. Immune cell infiltration in peritoneal adhesions of WT and STING‐KO mice Figure S10. STING‐mediated cytosolic DNA‐sensing pathway as new pathogenic mechanism in peritoneal damage Table S1. Demographic and clinical characteristics of control and PD patients Table S2. Postsurgical adhesion mouse scoring scheme Table S3. Predesigned assays used for qPCR Table S4. DEGs on peritoneum from CHX‐treated mice versus control mice [file PATH-267-196-s001.docx]

**STING inhibition alleviates experimental peritoneal damage: potential therapeutic relevance for peritoneal dialysis**

V Marchant *et al. J Pathol* <https://doi.org/10.1002/path.6462>

**Supplementary materials and methods**

**Supplementary material, Figures S1–S10**

**Supplementary material, Tables S1–S4**

*Reference numbers refer to the main text list.

**Supplementary materials and methods**

**Ethics approval**

Experiments on peritoneal biopsies from patients were performed according to the Declaration of Helsinki [27] and the STROBE reporting guidelines [28]. Written informed consent was approved by the Ethics Committee of Hospital Universitario La Paz, Madrid, Spain (HULP PI-4600; Ref. 07/253477.9/21) and obtained from all patients prior to sample obtention.

All animal procedures were performed according to the European Community guidelines for the care and use of laboratory animals, with prior approval by the Animal Ethics Committee of the IIS-FJD and Comunidad Autónoma de Madrid (PROEX 242.2/21).

**Human samples**

Formalin-fixed, paraffin-embedded (FFPE) peritoneal biopsies were used for STING immunostaining as described in what follows, in a case-control study design. Control biopsies were obtained from hemodialysis ESKD patients at the time of kidney transplantation or predialysis patients at the time of catheter insertion for PD (control ESKD group). Case biopsies were obtained from different ESKD patients on PD at the time of catheter removal in the kidney transplantation (ESKD-PD group). Demographic and clinical characteristics of patients are shown in supplementary material, Table S1. No patients were treated with immune suppressors.

**Animals**

C57BL/6J WT and C57BL/6J-STING1gt/J (systemic STING deficiency; STING-KO) male and female mice (8–12 weeks old) were obtained from Charles River Laboratories Spain (Barcelona, Spain) and maintained at the IIS-Fundación Jiménez Díaz animal facilities, with free access to food and water, normal light/dark cycles, and under specific pathogen-free conditions. Their wellbeing was monitored by the animal facility managers and researchers. All animal experiments were performed according to the ARRIVE reporting guidelines for the care and use of laboratory animals [31]. In brief, the experiments and sample size calculation were conducted following the 3Rs (replacement, reduction, and refinement) to promote welfare of animals used in research and to contribute to the improvement of scientific results. There were no previous criteria for including or excluding animals during the experiments and no exclusion of data points was made during the analysis. Mice were randomly distributed in different experimental groups in a blinded fashion.

**Chronic PDF exposure mouse models**

Two chronic PDF exposure models previously developed in our laboratory were used: one in nonuremic and the other in uremic/kidney insufficient 12- to 14-week-old female C57BL/6 mice (Charles River Laboratories, Barcelona, Spain). In brief, PDF (Stay Safe, lactate-buffered, 4.25% glucose; Fresenius Medical Care, Bad Homburg, Germany) infusion was performed using a peritoneal PD catheter connected to a subcutaneous mini access port, as previously described [30]. Nonuremic mice were daily infused with PDF (*n* = 6) or saline solution (0.9% NaCl; control, *n* = 6) for 30 days. Uremia and kidney insufficiency were induced in mice by 5/6 nephrectomy, as previously described [30]. Uremic mice received PDF (*n* = 6) or saline solution (control, *n* = 6) daily for 60 days. At the end of each model, mice were euthanized and biological samples were collected. FFPE parietal peritoneal tissue samples from mice were used for STING immunodetection, as described in what follows.

**CHX-induced peritoneal damage mouse models**

Peritoneal damage was induced in C57BL6/J male mice by daily i.p. injections of 0.1% CHX dissolved in saline solution at a volume of 10 ml/kg body weight, as previously described [93]. CHX was delivered daily at different time periods, as needed. To evaluate the progression of the CHX-induced peritoneal damage, mice were noninjected (*n* = 6, control group) or injected with CHX for 3, 10, or 30 days (*n* = 6, each group). Subsequently, two different CHX exposure models were used to evaluate the effect of STING genetic depletion. First, a 10-day-CHX exposure model was conducted in WT and STING-KO mice to evaluate inflammation (WT control group: *n* = 6; WT CHX group: *n* = 8; STING-KO control group: *n* = 4; and STING-KO CHX group: *n* = 4). Second, a 30-day-CHX exposure model was conducted in WT and STING-deficient (STING-KO) mice to evaluate fibrosis (WT control group: *n* = 5; WT CHX group: *n* = 8; STING-KO control group: *n* = 5; and STING-KO CHX group: *n* = 9). In addition, a 10-day-CHX infusion model was performed in parallel with the administration of the STING inhibitor C-176 (750 nmol/0.2 ml/mouse, dissolved in corn oil as vehicle; HY-112906 MedChemExpress, Monmouth Junction, NJ, USA), vehicle (alone or together with CHX), or CHX alone. The experimental groups of this model were as follows: control (*n* = 6), vehicle (*n* = 4), CHX (*n* = 6), CHX + vehicle (*n* = 6), and CHX + C-176 (*n* = 7). Mice from all CHX models were euthanized, peritoneal lavages were obtained, and parietal peritoneal tissue samples were collected according to the specific requirements for the subsequent analyses.

**Postsurgical intra-abdominal adhesions mouse model**

A standard surgical protocol was employed to perform the intra-abdominal adhesion model in 10- to 12-week-old C57BL6/J WT (*n* = 5) and STING-KO (*n* = 6) male mice, as previously described [40]. In brief, mice were anaesthetized with inhaled isoflurane during surgery. After the abdominal cavity was accessed, three IBs were made on the left side of the peritoneum by taking 3 mm of peritoneal parietal tissue with a hemostatic forceps and ligating the base of each segment with 4–0 silk sutures. In addition, the cecum was isolated and gently rubbed with sterile cotton swabs to induce injury in the visceral peritoneum and promote adhesions. After the procedure, the incision was closed, and mouse recovery was monitored. Five days after surgery, the operated mice and control WT (*n* = 5) and STING-KO (*n* = 6) mice without surgery were euthanized and the adhesions formed on IBs from operated mice were quantified and scored as previously described [40]. The extent (grade) and the quality (tenacity) of adhesions were scored for each IB with a point scale (supplementary material, Table S2). After examination, the parietal peritoneal tissue (IB and non-IB tissue) samples were collected according to the specific requirements for subsequent analyses.

***S. epidermidis*-induced peritonitis mouse model**

To induce bacterial peritonitis, 8- to 10-week-old WT (*n* = 6) and STING-KO (*n* = 4) female mice were i.p. injected with a single dose of live *Staphylococcus epidermidis* (×10^8^ CFU/mouse), as previously described [44,45]. WT (*n* = 6) and STING-KO (*n* = 6) mice without treatment were used as controls. Live *S. epidermidis* inoculum was prepared based on previously established procedures [45]. Mice were euthanized 72 h after bacterial injection, and parietal peritoneal tissue samples were collected according to the specific requirements for subsequent analyses.

**Transcriptomic analyses**

The RNA-seq study was carried out using peritoneal tissue samples from C57BL6/J male mice i.p. injected with 0.1% CHX daily for 10 days (*n* = 4) and noninjected mice as control (*n* = 4). RNA extraction, library preparation, and sequencing were performed in Fundación Parque Científico de Madrid (FPCM). In brief, mechanical disruption in a TissueLyser homogenizer (Qiagen, Hilden, Germany) and RNeasy Mini Kit (Qiagen), including on-column DNase treatment, were used for total RNA extraction from parietal peritoneal tissues, following the manufacturer’s protocol. Total RNA quality and concentration were assessed on 4200 TapeStation (Agilent Technologies, Santa Clara, CA, USA) using an RNA ScreenTape, confirming all samples had RIN > 7. Then, 500 ng of total RNA from each sample was used as input for library preparation with NEBNext Ultra II Directional RNA Library Prep Kit (New England Biolabs, Ipswich, MA, USA), following the manufacturer’s protocol for Poly(A) mRNA. The ultimately obtained libraries were validated and quantified by TapeStation and an equimolecular pool was made, purified using AMPure XP beads (Beckman Coulter, Brea, CA, USA) and titrated by PicoGreen. The library pool was sequenced on a NextSeq P4 flowcell (Illumina, San Diego, CA, USA), where clusters were formed and sequenced in a 1×75 single-read sequencing run on a NextSeq 2000 sequencer (Illumina). Read cleaning was performed with PrinSeq-lite (version 0.20.4). Mapping and alignments to the mouse genome were made with TopHat (version 2.1.1). Differential expression analysis and statistics were carried out with CuffDiff from Cufflinks (version 2.2.1). All software tools used are integrated in the GPRO Suite (Biotechvana, Valencia, Spain) [94]. The obtained RNA-seq data sets are available from the Gene Expression Omnibus repository under the following accession number: GSE282440 <https://www.ncbi.nlm.nih.gov/geo/query/acc.cgi?acc=GSE282440>. Data are reported following the MINSEQE guidelines [95].

**Functional enrichment analyses**

DEGs found in CHX-exposed mice versus control mice (obtained from the transcriptomic analysis) were used to perform a series of functional enrichment analyses using the following web tools and databases. Heatmapping and hierarchical clustering of DEGs were carried out using the ClustVis web tool (https://biit.cs.ut.ee/clustvis/; accessed 27 August 2024) [96]. For hierarchical clustering, rows were centered and unit variance scaling was applied to rows. Both rows and columns were clustered using correlation distance and average linkage. Functional enrichment analysis of cell type and transcription factors was performed through the Metascape web tool (https://metascape.org/; accessed 27 August 2024) [97], based on the Pattern Gene Database (PaGenBase) and TRRUST databases [98,99]. Signaling pathway enrichment analysis was carried out using the g:Profiler web server (https://biit.cs.ut.ee/gprofiler/gost; accessed 27 August 2024) [100] based on the Reactome and KEGG databases [35]. For KEGG enrichment, terms were filtered by including the word “pathway” to select only signaling pathways. Identification of upregulated DEGs on the KEEG cytosolic DNA-sensing pathway (mmu04623) was performed using the mapping tool of the KEGG database (https://www.kegg.jp/pathway/mmu04623) [35]. All functional enrichment analyses were performed for up- and downregulated DEGs separately.

**Cell Culture**

The human mesothelial cell line MeT-5A (ATCC, Rockville, MD, USA) was cultured in Earle’s M199 medium supplemented with 10% FBS (Gibco, Waltham, MA, USA), 20 mM HEPES (Gibco), and 100 U/ml penicillin and 100 μg/ml streptomycin (Gibco). Cells were grown at 37 °C in a humidified atmosphere with 5% CO_2_.

For *in vitro* assessment of mesothelial inflammation and MMT, MeT-5A cells were FBS-depleted for 24 h, pretreated with H-151 (1 μM) for 1 h when appropriate, and then stimulated with TGF-β1 (2 ng/ml) + IL-1 β (5 ng/ml) or TNF-α (5 ng/ml) for 24 h.

To obtain primary murine peritoneal macrophages, three C57BL6/J mice were i.p. injected with 3% thioglycolate to induce macrophage recruitment into the peritoneal cavity. After 72 h, macrophages were removed by washing the peritoneal cavity with saline solution. Macrophages were cultured in RPMI 1640 medium (Gibco) supplemented with 10% FBS (Gibco), 2 mM L-glutamine (Euroclone, Pero, Italy), 100 U/ml penicillin, and 100 μg/ml streptomycin (Gibco) and grown at 37 °C in a humidified atmosphere with 5% CO_2_. For the experiments, macrophages from each mouse were FBS-depleted for 24 h, pretreated with the STING inhibitor C-176 (MedChemExpress, Monmouth Junction, NJ, USA; HY-112906) at 1 μM concentration, dissolved in DMSO as the vehicle, and for 1 h or left untreated. The macrophages were then stimulated with LPS (1 µg/ml) for 6 h. After this time, the culture medium was renewed, and 24 h later, activated macrophage-conditioned medium (AMCM) was obtained and stored. The AMCM was pooled for each of the two experimental conditions, obtaining control AMCM (AMCM from non-pretreated macrophages) and C-176-AMCM (AMCM from macrophages pretreated with C-176) pools. Then, in three independent experiments, cultured MeT-5A cells (FBS-depleted for 24 h) were incubated for 48 h in the presence of control AMCM or C-176-AMCM and MeT-5A medium (M199), at a 1:1 proportion. MeT-5A cells incubated with RPMI medium, instead of AMCM, and vehicle were used as control condition. After the experiments, total RNA was isolated and gene expression of the MCs was evaluated.

**Histological and immunohistochemical analyses**

Peritoneal tissues collected from mice and patients (biopsies) were fixed in 4% formaldehyde, embedded in paraffin, and cut in 4-μm tissue sections for Masson’s trichrome staining (Bio-Optica, Milano, Italy), immunohistochemistry (IHC), or immunofluorescence (IF) studies. These experiments were performed and evaluated without investigators knowing the experimental group to which each sample corresponds (double-blind method).

For IHC, tissue sections were deparaffined and hydrated, and antigen retrieval was carried out using a sodium citrate buffer (10 mM. pH 6 or 9) on a PT Link system (DAKO, Glostrup, Denmark). Endogenous peroxidase blockade was done using 3% hydrogen peroxide (Millipore, Burlington, MA, USA) and protein blockade using a casein-blocking solution (Vector Laboratories, Newark, CA, USA). Then primary antibodies were incubated overnight at 4 °C, followed by incubation with biotinylated secondary antibodies (anti-rabbit or anti-rat, 1:200) and avidin-biotin complex (ABC, Vector Laboratories). Signal was detected using 3,3-diaminobenzidine (DAB) chromogen and substrate solution (Abcam, Cambridge, UK). Finally, slides were counterstained with hematoxylin (Merck, Darmstadt, Germany), dehydrated, and mounted with DPX (Merck). The following primary antibodies were used: rabbit anti-STING (1:500; Catalogue No.: 13647, Cell Signaling, Danvers, MA, USA), rat anti-F4/80 (1:50; MCA497, Bio-Rad, CA, USA), mouse anti-α-SMA (1:200; A2527, Sigma-Aldrich, Saint Louis, MO, USA), mouse anti-phospho(S536)-NF-κB p65 (1:50; sc-136548, Santa Cruz Biotechnology), rabbit anti-MPO (1:3000; A0398, Dako), rabbit anti-CD3 (1:100; A0452, Dako), mouse anti-CD4 (1:100; M7310, Dako), and rabbit anti-phospho(S40)-NRF2 (1:2,000; AB76026, Abcam).

For IF, deparaffination, hydration, and antigen retrieval of tissue sections were done as described earlier. Next, tissues were permeabilized with 0.2% Triton X-100/PBS, and protein blockade was performed using 10% rabbit or rat serum, as appropriate (diluted in 4% BSA in PBS). Then sections were incubated overnight at 4 °C, with the primary antibodies diluted in 1% serum (diluted in 4% BSA in PBS) followed by incubation with the fluorophore-conjugated secondary antibodies. The primary antibodies used were rabbit anti-STING (1:200; PA5-23381, Invitrogen, Waltham, MA, USA), rat anti-F4/80 (1:50; MCA497, Bio-Rad), and rat anti-CD31 (1:50; DIA-310-BA-2, Dianova, Hamburg, Germany), and the secondary antibodies were Alexa Fluor 633-conjugated anti-rabbit (1:200; Thermo Fisher Scientific, Waltham, MA, USA) and Alexa Fluor 488-conjugated anti-rat (1:200; A21208, Thermo Fisher Scientific). Nuclei were stained with DAPI (1:10.000; Sigma-Aldrich), and then stained sections were mounted with ProLong Gold antifade reagent (Invitrogen), and images were acquired using a fluorescence microscope (BX53, Olympus, Tokyo, Japan).

Peritoneal membrane thickness was determined by measuring the submesothelial zone width on Masson stain images using the ImageJ tool (five measurements/field in 5–10 fields per mouse, ×200 magnification), and IHC quantification was made by counting stained cells in 5–10 randomly chosen fields per mouse (×200 magnification).

**Flow cytometry analysis**

Cell suspensions obtained from peritoneal lavages were counted with a Scepter handheld automatic cell counter (Millipore). Then, 1×10^6^cells were stained with fluorochrome-conjugated mouse-specific antibodies against CD3, CD4, CD8, CD11b, F4/80, and Ly6G (BD Biosciences Pharmingen, San Diego, CA, USA) following the manufacturer’s protocol. An isotype control was used in each case to ensure staining specificity and avoid artifacts. Unstained cells were used as negative control for the analysis settings. Samples were analyzed in a BD FACS Canto II (BD Biosciences) flow cytometer, and data analyses were performed using FlowJo software version 10 (BD Biosciences).

**Protein-level studies**

Total protein from frozen peritoneal tissue and cultured cells were isolated by homogenization in a lysis buffer (T-PER, Thermo Fisher Scientific) with 10 μl/ml protease inhibitor cocktail, 0.2 mmol/l phenylmethylsulfonyl fluoride, and 0.2 mmol/l orthovanadate. Proteins were quantified using a Pierce BCA protein assay kit (Thermo Fisher Scientific) and then separated by electrophoresis using 8–10% polyacrylamide-SDS gels under reducing conditions for western blotting. Samples were then transferred onto polyvinylidene difluoride membranes (Thermo Fisher Scientific), blocked with 5% nonfat milk, and incubated overnight at 4 °C with the following primary antibodies: rabbit anti-STING (1:1,000; Catalogue No.: 13647, Cell Signaling), rabbit anti-TBK1 (1:1,000; Catalogue No.: 38066, Cell Signaling), rabbit anti-phospho(S172)-TBK1 (1:1,000; Catalogue No.: 5483, Cell Signaling), rabbit anti-IRF3 (1:1,000; Catalogue No.: 4302, Cell Signaling), rabbit anti-phospho(S396)-IRF3 (1:1,000; SAB4504031, Sigma-Aldrich), rabbit anti-phospho(S536)-NF-κB p65 (1:1,000; Catalogue No.: 3031, Cell Signaling), mouse anti-phospho(S32)-IκBα (1:1,000; sc-8404, Santa Cruz), and rabbit anti-fibronectin (1:5,000; AB2033, Millipore). Then the membranes were incubated with HRP-conjugated secondary antibodies (anti-rabbit or anti-mouse, 1:5,000). Loading controls were performed using a mouse anti-GAPDH antibody (1:5,000; CB1001, Millipore) and mouse α-tubulin (1:5,000; T5168, Sigma-Aldrich). Proteins on membranes were visualized using the chemiluminescence detection kit Immobilon Crescendo Western HRP substrate (Millipore) on the Amersham Imager 600 instrument (GE Healthcare, Chicago, IL, USA). Images were analyzed by densitometry using ImageJ, an image processing program written in Java (https://imagej.net/ij/; National Institutes of Health, Bethesda, MD, USA).

**Gene expression assays**

Total RNA was isolated from frozen peritoneal tissue and pelleted cultured cells by homogenization with TRItidy G (PanReac, Darmstadt, Germany), following the manufacturer’s protocol. Next, cDNA was synthesized using a High-Capacity cDNA Reverse Transcription Kit (Applied Biosystems, Waltham, MA, USA) using 2 µg of total RNA. Gene expression analysis was determined by quantitative PCR (qPCR) using the commercial master mix Premix Ex Taq (Takara, Otsu, Japan) and predesigned TaqMan-based qPCR assays for target and housekeeping genes (supplementary material, Table S3) and run on the 7500 Fast Real-Time PCR System and QuantStudio 3 thermocyclers (Applied Biosystems, Waltham, CA, USA). Relative expression levels were obtained normalizing the values by the expression of housekeeping genes using the 2^-ΔΔCt^ method [101]. The results were expressed as fold-change (*n*-fold) relative to control, and then the row *Z*-scores were calculated for each gene.

**Statistical analyses**

In most of the figures, the results are represented as mean ± SEM or median with upper and lower quartiles. The Shapiro–Wilk test was used to evaluate sample normality distribution. To assess statistical differences between groups, several tests were used based on data characteristics. For two-group comparisons, the F-test was used to check for homogeneity of variances; unpaired Student’s *t*-tests were conducted for normal samples (with or without Welch's correction depending on variance); and the Mann–Whitney test was used for samples without normal distribution. For comparisons involving more than two groups, Bartlett's test was used to assess homoscedasticity. Parametric one-way ANOVA followed by Fisher’s least significant difference (LSD) test was used for normally distributed homoscedastic samples. For heteroscedastic samples, Welch-corrected one-way ANOVA and Brown–Forsythe tests were followed by Welch's *t*-tests. A nonparametric Kruskal–Wallis test was applied for nonnormally distributed samples, followed by Dunn’s multiple-comparisons test. Two-way ANOVA followed by Fisher’s LSD was used for experiments involving two factors (treatment and genotype).

For cell culture experiments, paired comparisons were conducted using the repeated measures (RM) one-way ANOVA for normal data or the Friedman test for data not normally distributed, followed by Fisher's LSD or Dunn’s *post hoc* tests, respectively.

All statistical analyses and graphs were performed using GraphPad Prism 8.0 (GraphPad Software, San Diego, CA, USA). *P* values*<*0.05 were considered statistically significant.

**
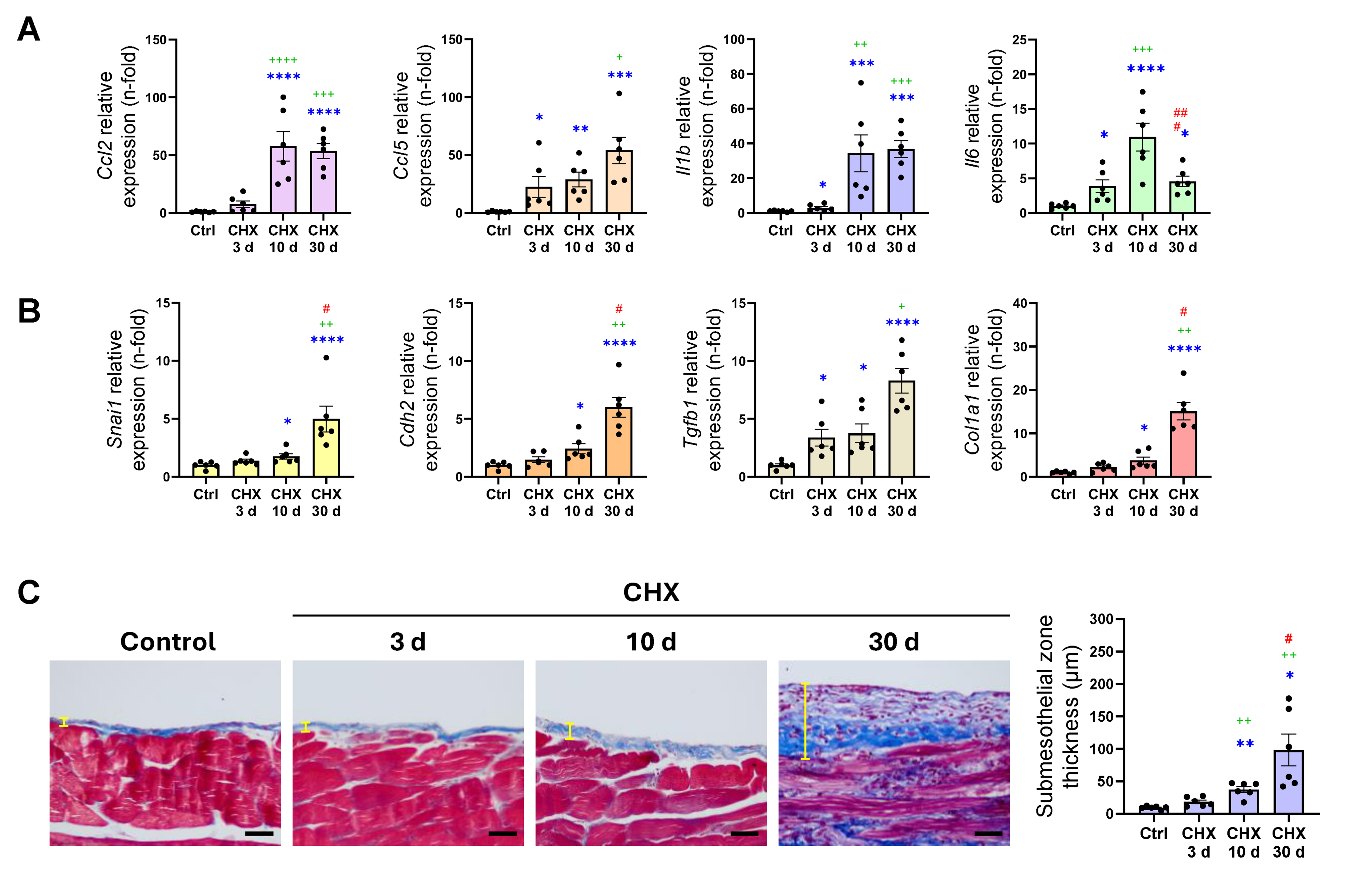
**

**Figure S1.** Time course of peritoneal damage induced by CHX in mice. Male C57BL/6J mice were daily i.p. injected with 0.1% CHX for 3, 10, or 30 days (d). (A and B) Relative gene expression of (A) proinflammatory chemokines and cytokines and (B) MMT and fibrosis markers analyzed by RT-qPCR from total RNA of parietal peritoneal tissue, using *Gapdh* as a housekeeping gene, and expressed as fold-change (*n*-fold) relative to control. (C) Peritoneal membrane thickness assessment. The figure shows Masson’s trichrome-stained parietal peritoneal tissue sections of representative mice from each group (left) and the corresponding quantification of submesothelial zone thickness (right). Yellow lines indicate width measured**.** Scale bar, 50 μm. Results are represented as mean ± SEM of *n* = 6 animals per group. **p* < 0.05, ***p* < 0.01, ****p* < 0.001, *****p* < 0.0001 versus control (Ctrl). +*p* < 0.05, ++*p* < 0.01, +++*p* < 0.001, ++++*p* < 0.0001 versus CHX 3 days; #*p* < 0.05, ###*p* < 0.001 versus CHX 10 days.

**
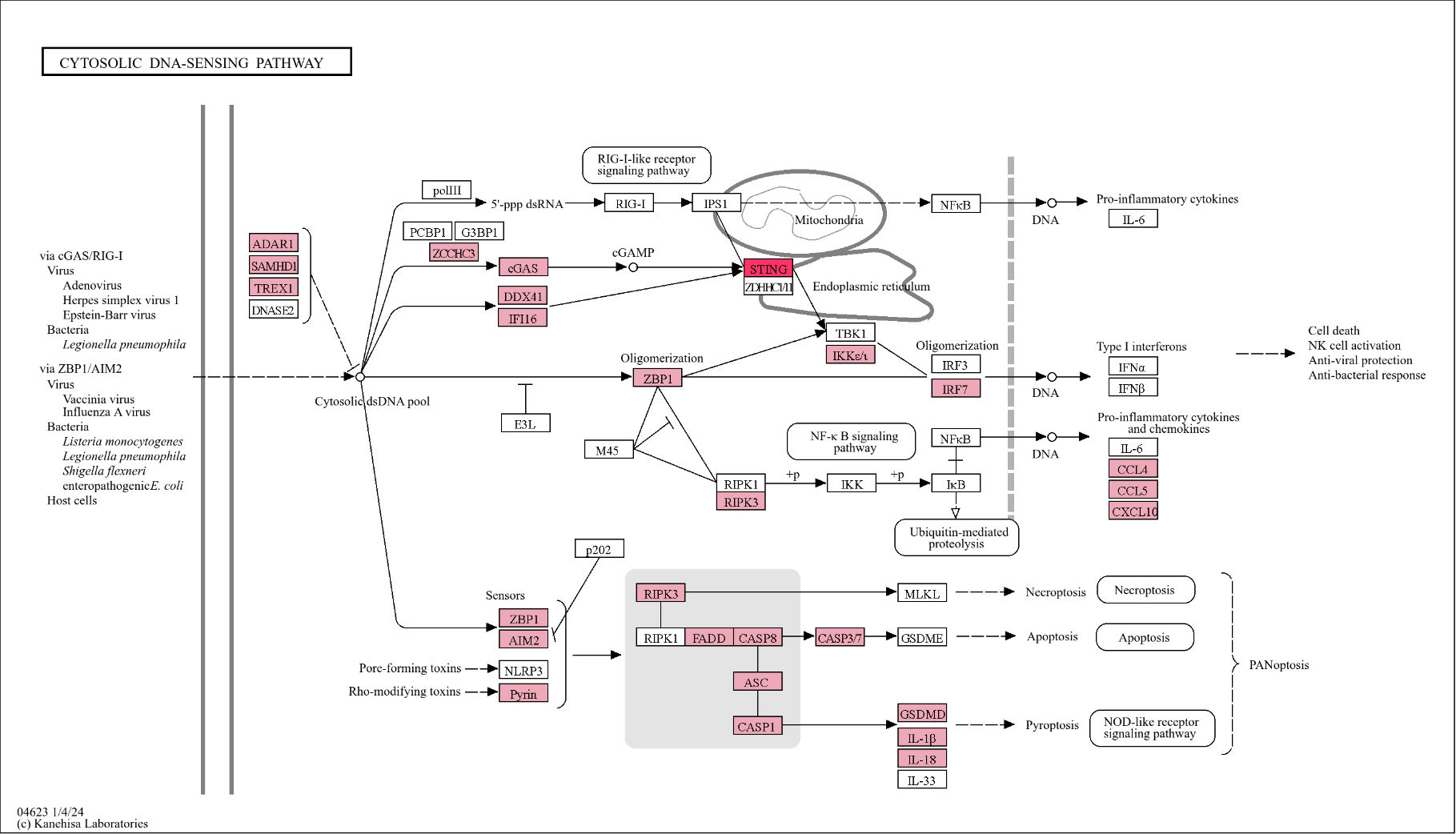
**

**Figure S2.** Upregulated genes of cytosolic DNA-sensing pathway in peritoneum of 10-day-CHX-exposed mice. This pathway map was generated using the KEGG website (https://www.kegg.jp/pathway/mmu04623), which enables gene highlighting and color customization but does not allow font modification. The code 04623 corresponds to the specific pathway, and the date “1/4/24” refers to the most recent revision of the map by the database curators at Kanehisa Laboratories. Kanehisa is also the corresponding author of reference [35].

Upregulated genes of cytosolic DNA-sensing pathway in peritoneum of 10-day-CHX-exposed mice. The figure shows the cytosolic DNA-sensing pathway (Mus musculus; mmu04623 https://www.kegg.jp/pathway/mmu04623) generated by the KEGG database (pathway revised on 1/4/24 by Kanehisa Laboratories) [35]. Genes of this pathway that were significantly upregulated (*q*-value < 0.05) in the transcriptomic analysis performed in the parietal peritoneal tissue of mice treated with 0.1% CHX for 10 days are highlighted in light pink. STING term is highlighted in red.

**
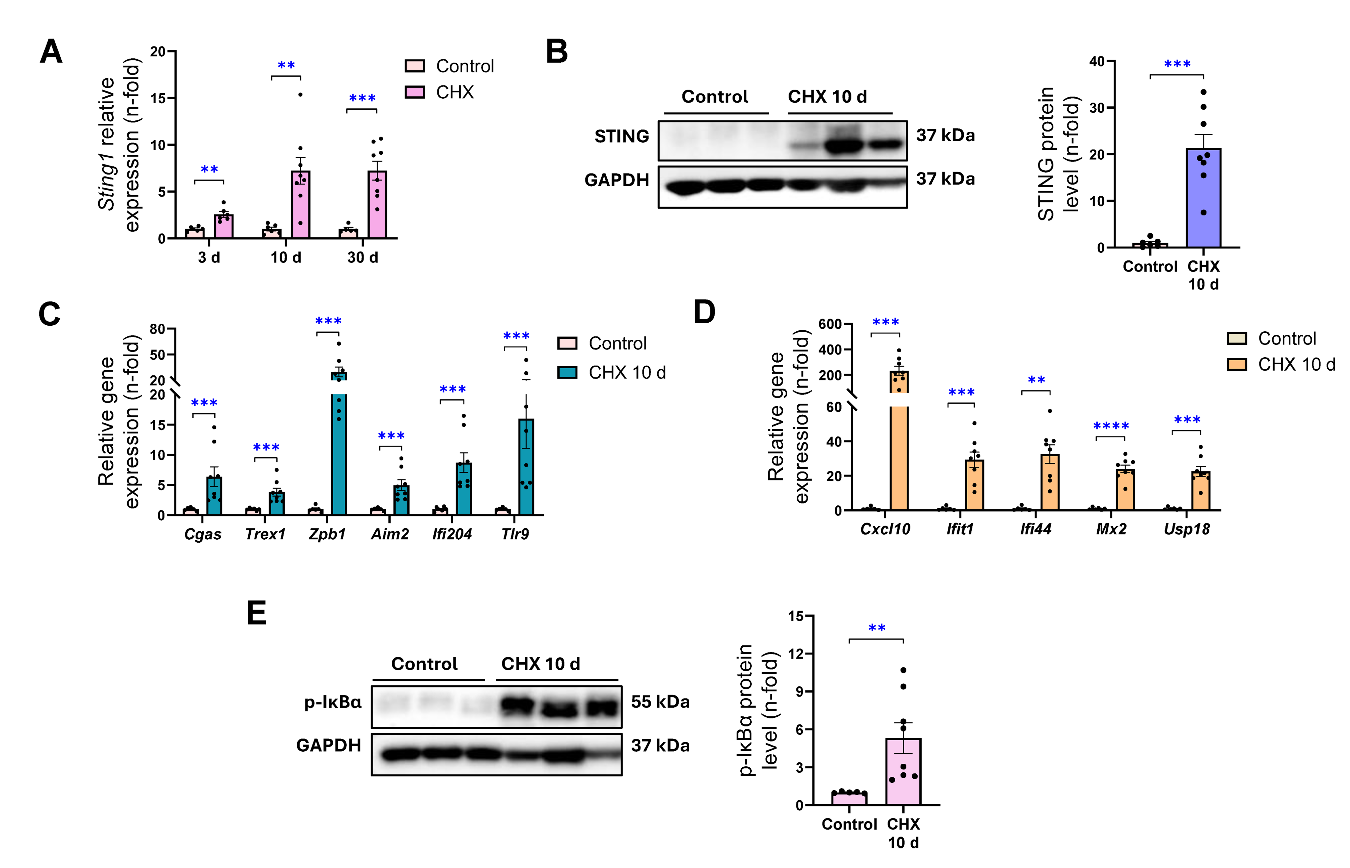
**

**Figure S3.** Expression of STING and other cytosolic DNA sensors in peritoneum of CHX-exposed mice. (A–E) Male C57BL/6J mice were daily i.p. injected with 0.1% CHX for 3 or 10 days. (A) Relative gene expression of *Sting1* in peritoneum of mice exposed to CHX for 3, 10, and 30 days. (B) Peritoneal STING protein levels in mice exposed to CHX for 10 days. (C) Relative expression of cytosolic DNA sensors in the peritoneum of mice exposed to CHX for 10 days. (D) Relative expression of IFN-stimulated genes in peritoneum of mice exposed to CHX for 10 days. (E) Peritoneal p-IκBα protein levels in mice exposed to CHX for 10 days. Relative gene expression was analyzed by RT-qPCR from total RNA of parietal peritoneal tissue using *Gapdh* as housekeeping gene. Protein levels were assessed by western blotting from total proteins of parietal peritoneal tissue using GAPDH as loading control. Results are expressed as *n*-fold compared to control and represented as mean ± SEM of five to eight animals per group. ***p* < 0.01, ****p* < 0.001, *****p* < 0.0001.


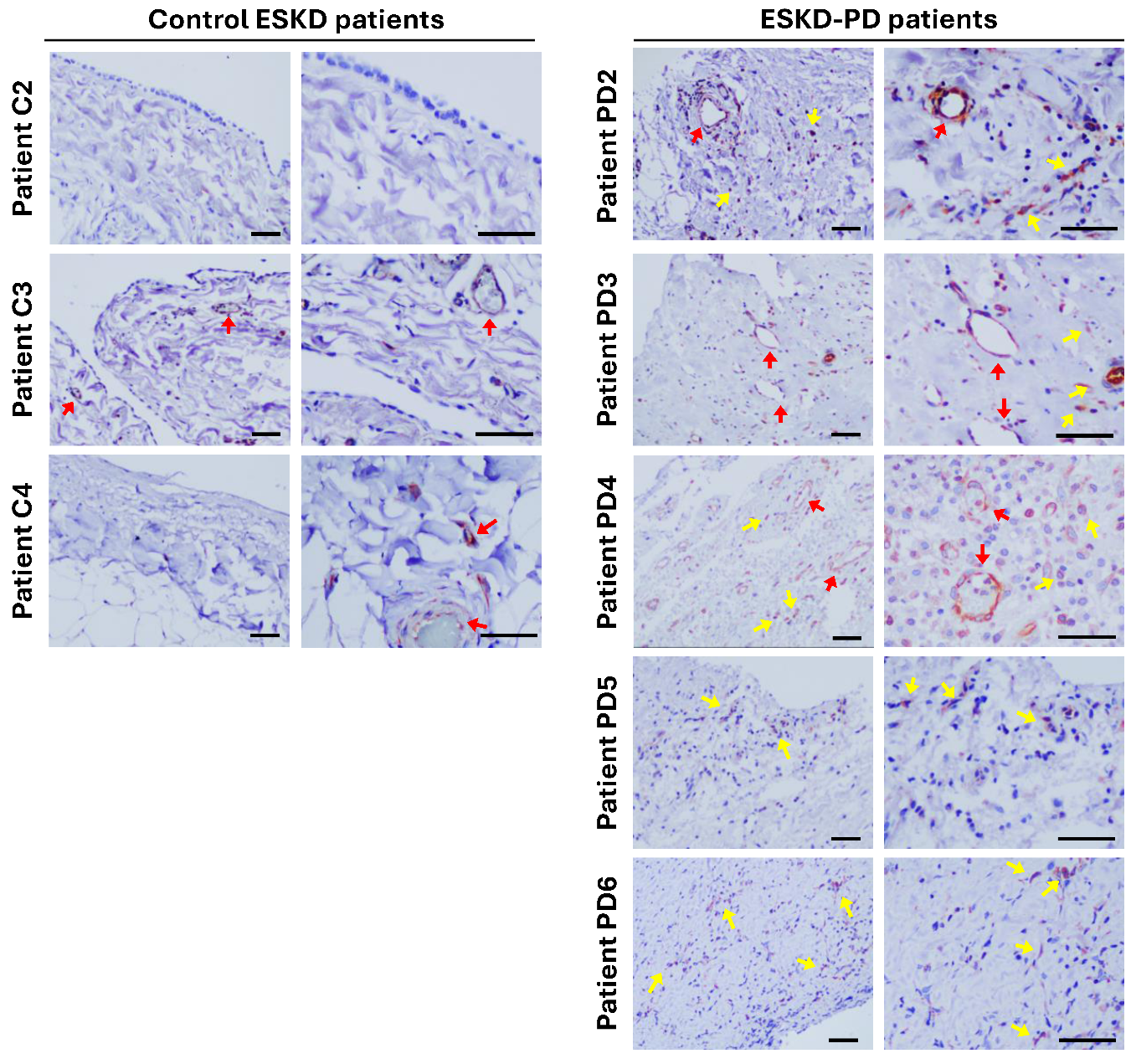


**Figure S4.** STING^+^ staining in peritoneal biopsies from PD patients. Immunohistochemical staining of STING sections of peritoneal biopsies from control ESKD and ESKD-PD patients. Scale bar, 50 μm. Red arrows indicate STING^+^ stain in endothelium; yellow arrows indicate STING^+^ stain in submesothelial zone (stroma).

**
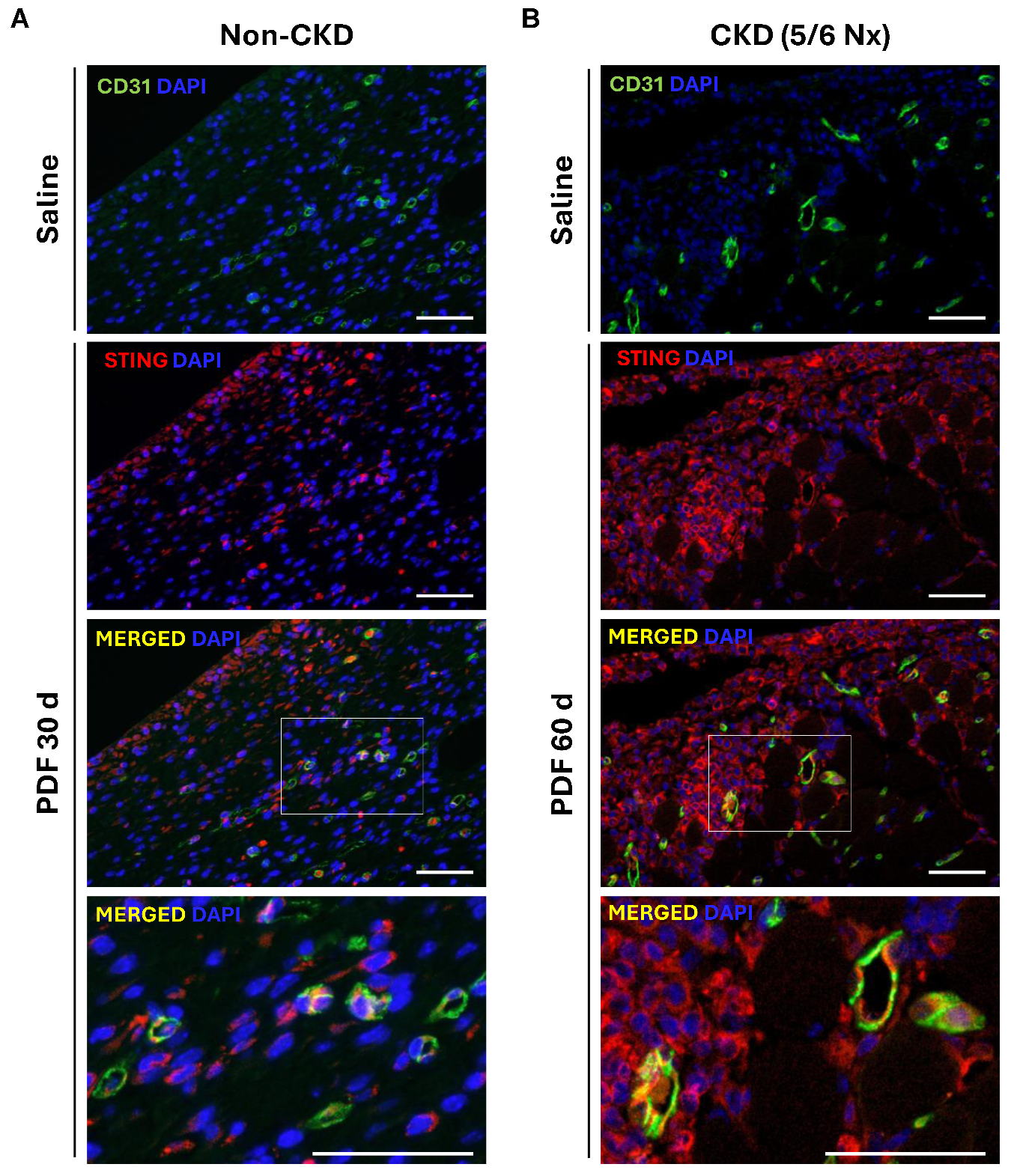
**

**Figure S5.** STING^+^ staining in peritoneal endothelium PDF-exposed mice. IF staining of STING (red) and CD31 (green) shows the presence of double-labeled CD31^+^STING^+^ (merged, yellow) endothelial cells in peritoneum of 30-day-PDF-exposed (A) and 60-day-PDF-exposed 5/6 nephrectomized (B) C57BL/6J female mice. Microscopy images correspond to a representative animal from each group. The lower panels display a magnified detail of merged image shown above. Scale bar, 50 μm. Nx: nephrectomy.

**
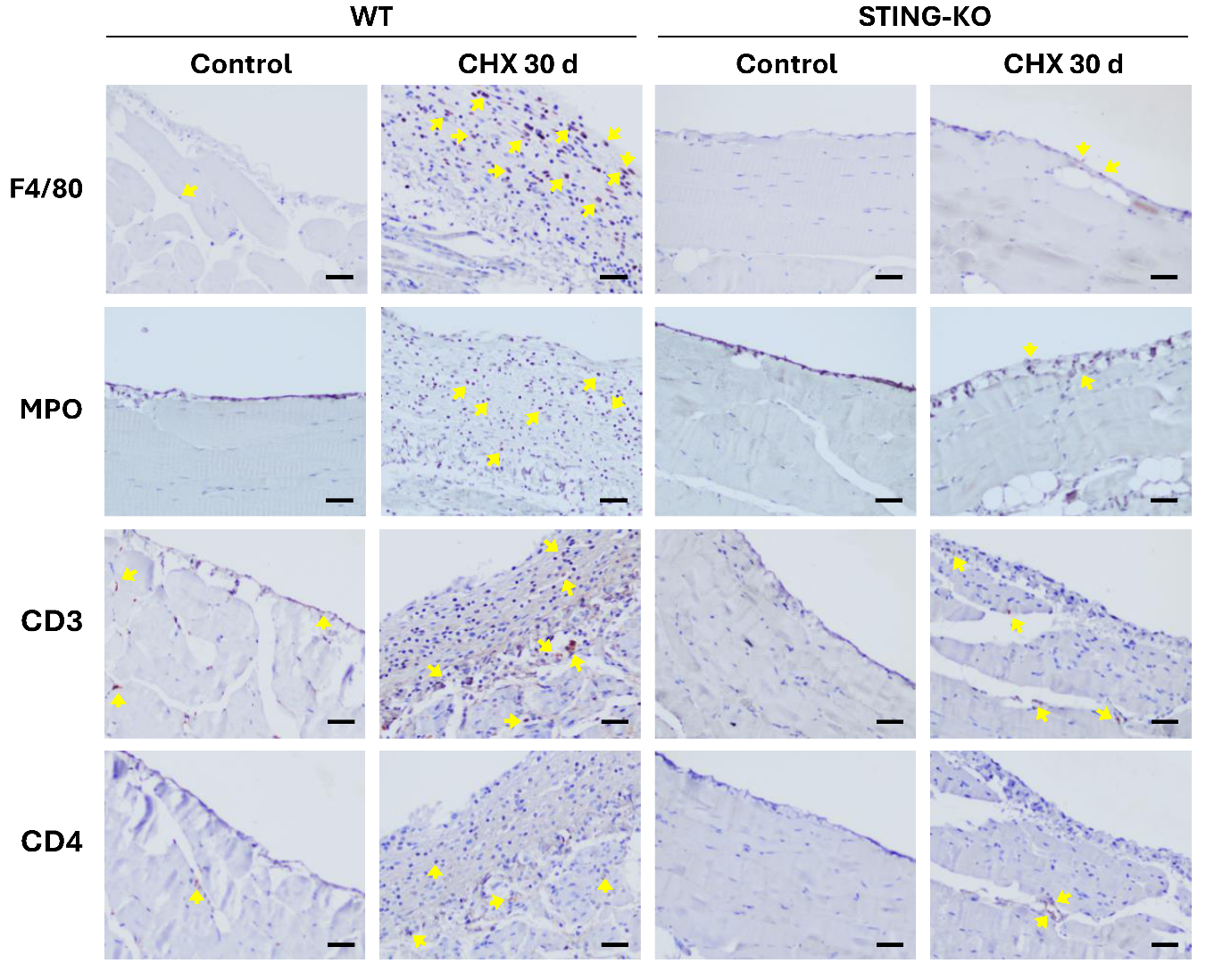
**

**Figure S6.** Inflammatory cell infiltration into the peritoneum of WT and STING-KO mice exposed to CHX for 30 days. Male C57BL/6J WT and STING-deficient (STING-KO) mice were daily i.p. injected with 0.1% CHX for 30 days. Parietal peritoneal tissue sections were used for IHC staining to identify inflammatory immune cell subsets using specific antibodies against the following markers: F4/80 (macrophages), myeloperoxidase (MPO, neutrophils), and CD3 and CD4 (T cells). Microscopy images correspond to a representative animal from each group. Yellow arrows indicate cells with positive staining. Scale bar, 50 μm.

**
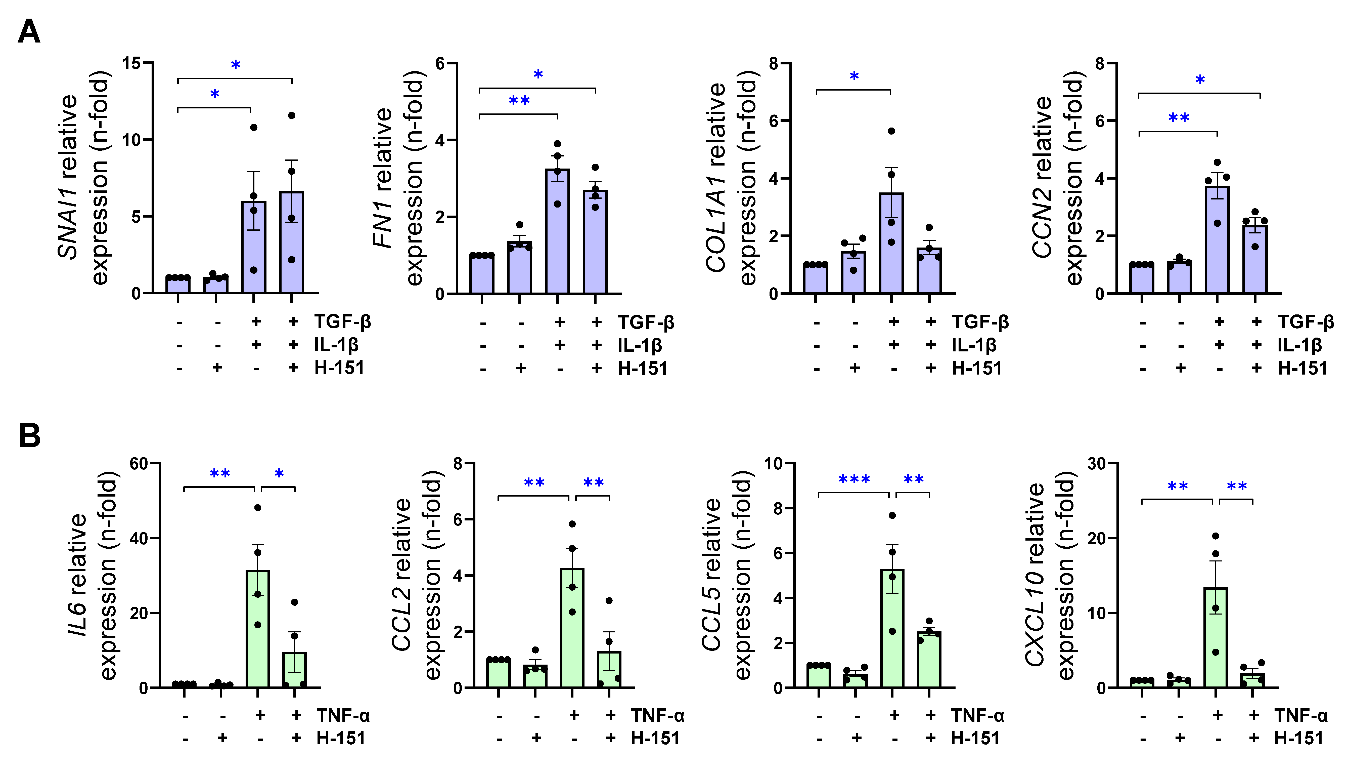
**

**Figure S7.** Pharmacological inhibition of STING in cultured mesothelial cells. Human MeT-5A mesothelial cells were pretreated with STING inhibitor H-151 (1 µM) for 1 h and then stimulated with recombinant TGF-β (2 ng/ml) and IL-1-β (5 ng/ml) (A) or TNF-α (5 ng/ml) (B) for 24 h. (A) Relative gene expression of MMT/fibrosis markers. (B) Relative gene expression of inflammatory markers. Relative gene expression levels were analyzed by RT-qPCR from total RNA, using *GAPDH* as housekeeping gene. Results are expressed as fold-change (*n*-fold) relative to control condition (first column) and represented as mean ± SEM of four independent experiments. **p* < 0.05, ***p* < 0.01, ****p* < 0.001, *****p* < 0.0001.

**
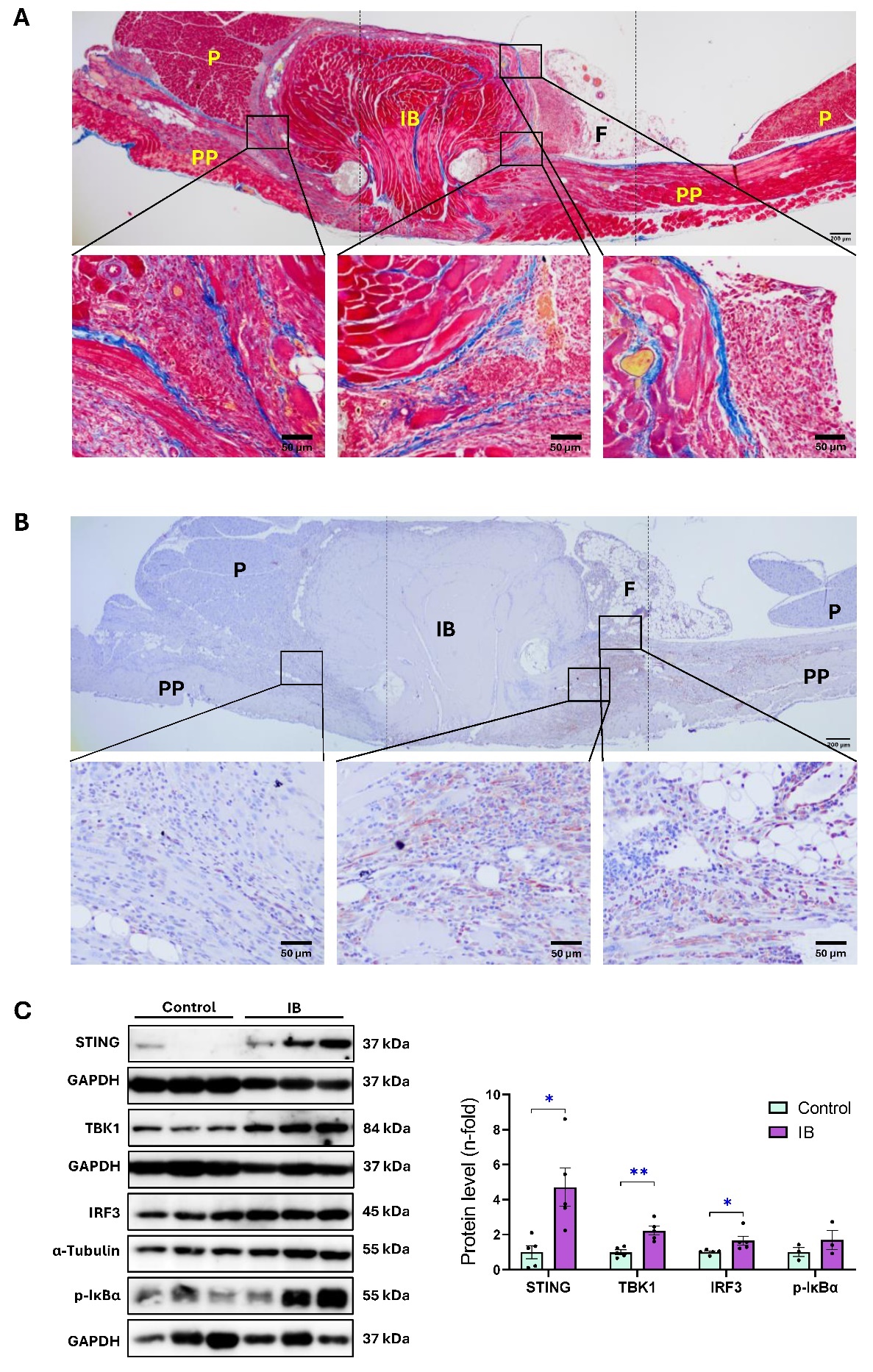
**

**Figure S8.** Expression of STING and downstream signaling mediators in postsurgical peritoneal adhesions. IBs were surgically generated in peritoneum of male C57BL/6J mice and formation of adhesions between peritoneal IBs and surrounding tissues was assessed 5 days after surgery. (A) Masson’s trichrome staining in sections of parietal peritoneal tissue containing IBs. Peritoneal adhesions of parietal peritoneum (PP) with neighbor organ and tissues like pancreas (P) and fat (F) are shown. Squared areas containing peritoneal adhesion interfaces are shown at higher magnitude below. (B) Immunohistochemical detection of STING in sections of parietal peritoneal tissue containing IBs. The figure shows STING^+^ cells of the peritoneal peritoneum based near peritoneal adhesions. Squared areas containing STING^+^ cells are depicted at a higher magnitude below. Dashed lines indicate boundaries between adjacent joined images. Microscopy images correspond to IB with adhesions with grade and tenacity scores equal to 5. Scale bars, 200 μm and 50 μm. (C) Protein levels of STING, TBK1, IRF3, and p-IκBα were assessed by western blot from total protein extracts of parietal peritoneal tissues using GAPDH or α-tubulin as loading control. Results are represented as fold-change (*n*-fold) relative to control group and expressed as mean ± SEM of five animals per group. **p* < 0.05, ***p* < 0.01.

**
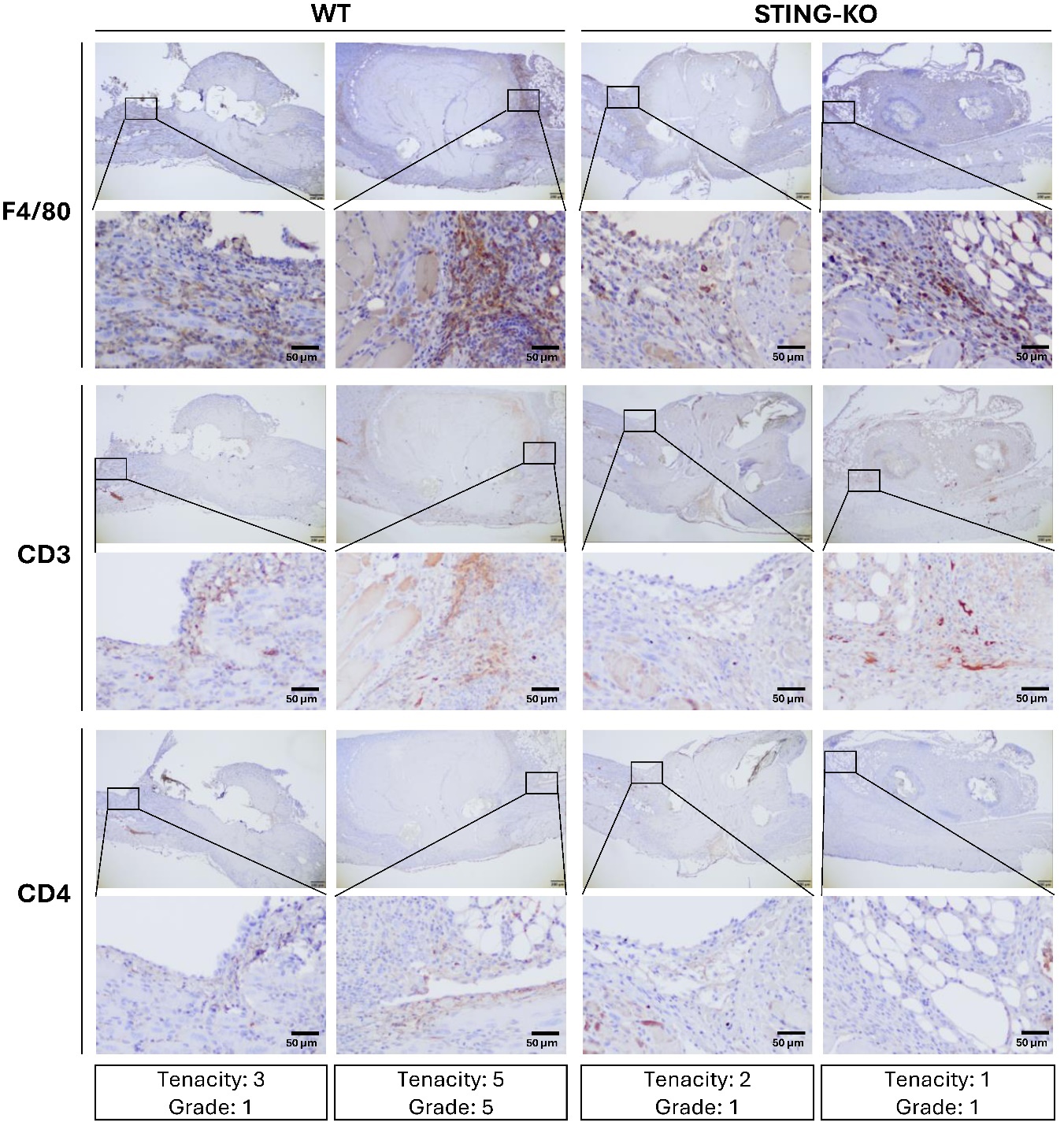
**

**Figure S9.** Immune cell infiltration in peritoneal adhesions of WT and STING-KO mice. Three IBs were surgically made in peritoneum of male C57BL/6J WT and STING-deficient (STING-KO) mice. The formation of adhesions on the IBs was assessed 5 days after surgery. Sections of parietal peritoneal tissue containing the IBs were used for immunohistochemical detection of F4/80^+^ macrophages and CD3^+^ and CD4^+^ T cells. Microscopy images correspond to two representative animals from each group. The squared areas are shown at higher magnification below every original micrograph. Scale bars, 200 µm and 50 µm.

**
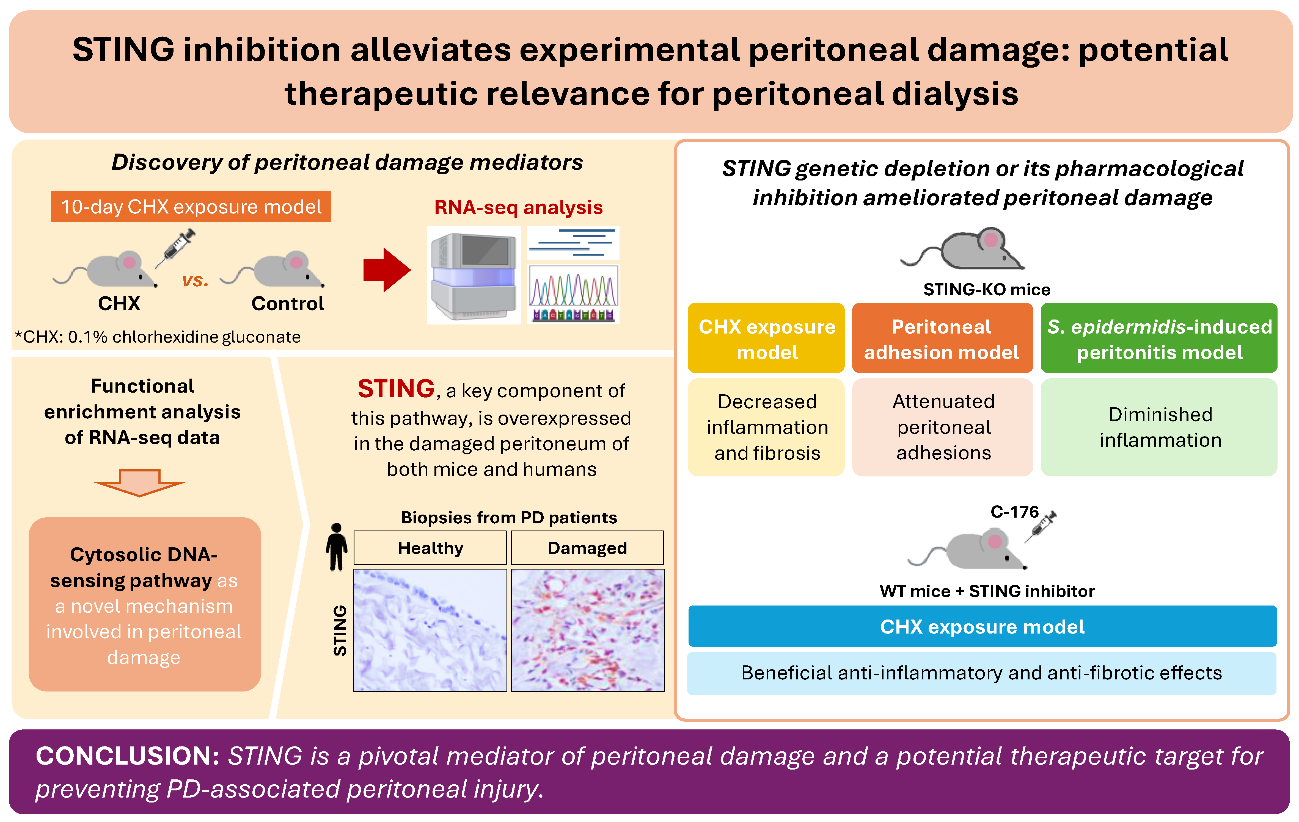
**

**Figure S10.** STING-mediated cytosolic DNA-sensing pathway as new pathogenic mechanism in peritoneal damage. RNA-seq of peritoneal tissue from mice exposed to 0.1% CHX for 10 days, compared to unexposed controls, identified the cytosolic DNA-sensing pathway as one of the most significantly upregulated pathways in experimental peritoneal injury. STING, a central mediator of this pathway, was found to be upregulated in the damaged peritoneum of both CHX-exposed mice and PD patients. Genetic deletion or pharmacological inhibition of STING-attenuated peritoneal injury across multiple murine models. These findings highlight STING as a promising therapeutic target for treatment of PD-associated peritoneal damage. Created using elements from BioRender ([https://www.biorender.com/](https://eur01.safelinks.protection.outlook.com/?url=https%3A%2F%2Fwww.biorender.com%2F&data=05%7C02%7CMcAteer-SCIED%40jpathol.org%7Cfdf6e84eed40453b261008ddbfc86316%7Ce87674224a79483e851b9dede9600424%7C0%7C0%7C638877589644288238%7CUnknown%7CTWFpbGZsb3d8eyJFbXB0eU1hcGkiOnRydWUsIlYiOiIwLjAuMDAwMCIsIlAiOiJXaW4zMiIsIkFOIjoiTWFpbCIsIldUIjoyfQ%3D%3D%7C0%7C%7C%7C&sdata=LZwvpsIBzAJKzPvFKuRq39CmRCj0Qs73M3SI3EdzeoQ%3D&reserved=0)).

**Table S1.** Demographic and clinical characteristics of control and PD patients.

| Group | Patient | Sex | Age (years) | Time in PD (months) | Cause of biopsy | Basal disease | Peritonitis | MTC Urea (ml/min) | MTC Cr (ml/min) | UF glucose 3.86% (ml) | D/P Cr |
| --- | --- | --- | --- | --- | --- | --- | --- | --- | --- | --- | --- |
| Control | C1 | Male | 59 | - | Transplant | CPN | No | - | - | - | - |
| ESKD | C2 | Male | 60 | - | Transplant | NI | No | - | - | - | - |
|  | C3 | Male | 57 | - | Transplant | DM | No | - | - | - | - |
|  | C4 | Female | 72 | - | Catheter insertion | CGN | No | - | - | - | - |
| ESKD- | PD1 | Male | 72 | 36 | Transplant | CGN | No | 22.7 | 6.7 | 666 | 0.6 |
| PD | PD2 | Male | 35 | 12 | Transplant | CGN | No | 19 | 4 | 1,360 | 0.64 |
|  | PD3 | Female | 55 | 21 | Transplant | CGN | No | 25.1 | 8.9 | 600 | 0.68 |
|  | PD4 | Female | 65 | 25 | Transplant | NI | No | 13.2 | 4.6 | 800 | 0.61 |
|  | PD5 | Male | 25 | 52 | Transplant | CGN | No | 19.3 | 6.6 | 850 | 0.81 |
|  | PD6 | Female | 50 | 37 | Transplant | NI | No | 22.1 | 10.8 | 310 | 0.67 |

C: control; PD: peritoneal dialysis; DM: diabetes mellitus; NI: not informed; CPN: chronic pyelonephritis; CGN: crescentic glomerulonephritis; MTC: mass transfer coefficient; Cr: creatinine; UF: ultrafiltration in 4 h; D/P: dialysate-to-plasma ratio.

**Table S2.** Postsurgical adhesion mouse scoring scheme.

| Grade | Tenacity |
| --- | --- |
| (0) 0% | (0) No adhesion |
| (1) <25% | (1) Adhesion fell apart |
| (2) 25–50% | (2) Adhesion lysed with traction |
| (3) 51–75% | (3) Adhesion lysed with blunt dissection |
| (4) >75% | (4) Adhesion lysed with sharp dissection |
|  | (5) Adhesion between abdominal wall and organs |

*Grade is the extent of IB that is covered by the adhesion, expressed as percentage.

**Table S3.** Predesigned assays used for qPCR.

| Specie | Gene | Gene type | Assay ID | Company |
| --- | --- | --- | --- | --- |
| Mouse | *Acta2* | Target | Mm.PT.58.16320644 | Integrated DNA Technologies, Newark, NJ, USA |
| Mouse | *Arg1* | Target | Mm.PT.58.8651372 | Integrated DNA Technologies |
| Mouse | *Arg2* | Target | Mm.PT.58.31712462 | Integrated DNA Technologies |
| Mouse | *Cat* | Target | Mm.PT.58.12825133 | Integrated DNA Technologies |
| Mouse | *Ccl2* | Target | Mm.PT.58.42151692 | Integrated DNA Technologies |
| Mouse | *Ccl5* | Target | Mm.PT.58.43548565 | Integrated DNA Technologies |
| Mouse | *Ccl8* | Target | Mm01297183_m1 | Applied Biosystems, Waltham, MA, USA |
| Mouse | *Ccl19* | Target | Mm00839966_g1 | Applied Biosystems |
| Mouse | *Cd163* | Target | Mm00474091_m1 | Applied Biosystems |
| Mouse | *Cdh2* | Target | Mm.PT.58.12378183 | Integrated DNA Technologies |
| Mouse | *Col1a1* | Target | Mm.PT.58.7562513 | Integrated DNA Technologies |
| Mouse | *Col1a2* | Target | Mm.PT.58.5206680 | Integrated DNA Technologies |
| Mouse | *Cxcl1* | Target | Mm04207460_m1 | Applied Biosystems |
| Mouse | *Cxcl10* | Target | Mm.PT.58.43548565 | Integrated DNA Technologies |
| Mouse | *Hmox1* | Target | Mm.PT.58.8600055 | Integrated DNA Technologies |
| Mouse | *Il1b* | Target | Mm.PT.58.41616450 | Integrated DNA Technologies |
| Mouse | *Il10* | Target | Mm01288386_m1 | Applied Biosystems |
| Mouse | *Il6* | Target | Mm.PT.58.10005566 | Integrated DNA Technologies |
| Mouse | *Ifi44* | Target | Mm.PT.58.12162024 | Integrated DNA Technologies |
| Mouse | *Ifit1* | Target | Mm.PT.58.32674307 | Integrated DNA Technologies |
| Mouse | *Ifna1* | Target | Mm.PT.58.43426930.g | Integrated DNA Technologies |
| Mouse | *Ifnb1* | Target | Mm.PT.58.30132453.g | Integrated DNA Technologies |
| Mouse | *Ifng* | Target | Mm.PT.58.41769240 | Integrated DNA Technologies |
| Mouse | *Mx2* | Target | Mm.PT.58.29837402 | Integrated DNA Technologies |
| Mouse | *Nfe2l2* | Target | Mm.PT.58.29108649 | Integrated DNA Technologies |
| Mouse | *Nox1* | Target | Mm.PT.58.29694286 | Integrated DNA Technologies |
| Mouse | *Nox4* | Target | Mm.PT.58.8820983 | Integrated DNA Technologies |
| Mouse | *Oasl2* | Target | Mm01201449_m1 | Integrated DNA Technologies |
| Mouse | *Snai1* | Target | Mm00441533_g1 | Applied Biosystems |
| Mouse | *Sod1* | Target | Mm.PT.58.12368303 | Integrated DNA Technologies |
| Mouse | *Sod2* | Target | Mm.PT.58.14276358 | Integrated DNA Technologies |
| Mouse | *Sting1* | Target | Mm.PT.58.12798185 | Integrated DNA Technologies |
| Mouse | *Tgfb1* | Target | Mm.PT.58.11254750 | Integrated DNA Technologies |
| Mouse | *Usp18* | Target | Mm.PT.58.28965870 | Integrated DNA Technologies |
| Mouse | *Ppia* | Housekeeping | Mm.PT.39a.2.gs | Integrated DNA Technologies |
| Mouse | *Gapdh* | Housekeeping | Mm.PT.39a.1 | Integrated DNA Technologies |
| Human | *CCL2* | Target | Hs.PT.58.45467977 | Integrated DNA Technologies |
| Human | *CCL5* | Target | Hs.PT.58.1724551 | Integrated DNA Technologies |
| Human | *CDH2* | Target | Hs.PT.58.26024443 | Integrated DNA Technologies |
| Human | *CXCL10* | Target | Hs.PT.58.3790956.g | Integrated DNA Technologies |
| Human | *FN1* | Target | Hs.PT.58.40005963 | Integrated DNA Technologies |
| Human | *GREM1* | Target | Hs.PT.58.21084086 | Integrated DNA Technologies |
| Human | *SNAI1* | Target | Hs.PT.58.2984401 | Integrated DNA Technologies |
| Human | *TGFB1* | Target | Hs.PT.58.39813975 | Integrated DNA Technologies |
| Human | *USP18* | Target | Hs.PT.58.25205207 | Integrated DNA Technologies |
| Human | *GAPDH* | Housekeeping | Hs.PT.39a.22214836 | Integrated DNA Technologies |

**Table S4.** DEGs on peritoneum from CHX-treated mice versus control mice. The table shows the 1,050 genes with a |Log_2_(FC)| ≥ 2 and *q*-value < 0.05 found in the transcriptomic analysis.

| Gene  name | Log_2_ (FC) | *q*-value | Gene  name | Log_2_ (FC) | *q*-value | Gene name | Log_2_ (FC) | *q*-value |
| --- | --- | --- | --- | --- | --- | --- | --- | --- |
| *Cd300e* | + | 0.0004 | ***Saa3*** | 6.21 | 0.0004 | ***Mx2*** | 4.98 | 0.0004 |
| *Gjb4* | + | 0.0004 | ***A530064D06Rik*** | 6.21 | 0.0291 | ***Cd84*** | 4.97 | 0.0004 |
| *Tarm1* | + | 0.0004 | ***Gm12250*** | 6.16 | 0.0123 | ***Ifi47*** | 4.97 | 0.0023 |
| *Saa2* | + | 0.0004 | ***Lgals7*** | 6.14 | 0.0004 | ***Ifi204*** | 4.96 | 0.0004 |
| *Prf1* | + | 0.0004 | ***Irf7*** | 6.12 | 0.0004 | ***Hk3*** | 4.94 | 0.0004 |
| *Bcl2a1a* | + | 0.0004 | ***Apol9a*** | 6.06 | 0.0004 | ***Vcam1*** | 4.89 | 0.0004 |
| *4930430E12Rik* | + | 0.0004 | ***Mt2*** | 6.04 | 0.0004 | ***Gm20559*** | 4.87 | 0.0004 |
| *Gm5150* | + | 0.0004 | ***Rsad2*** | 6.01 | 0.0004 | ***Mpeg1*** | 4.87 | 0.0004 |
| *Sirpb1b* | + | 0.0004 | ***Lgals3*** | 6.00 | 0.0004 | ***Lilra6*** | 4.85 | 0.0004 |
| *Rgs1* | + | 0.0004 | ***Gm17455*** | 5.97 | 0.0258 | ***Aif1*** | 4.84 | 0.0004 |
| *Il1b* | + | 0.0004 | ***Slurp1*** | 5.92 | 0.0385 | ***Rpp25*** | 4.84 | 0.0004 |
| *Sirpb1c* | + | 0.0004 | ***Clec4d*** | 5.91 | 0.0004 | ***Serpina3n*** | 4.83 | 0.0004 |
| *Gzmb* | + | 0.0004 | ***Trib3*** | 5.89 | 0.0004 | ***Usp18*** | 4.83 | 0.0004 |
| *I830127L07Rik* | + | 0.0004 | ***Gng2*** | 5.89 | 0.0004 | ***Ms4a6b*** | 4.83 | 0.0004 |
| *Fpr1* | + | 0.0004 | ***Oasl1*** | 5.87 | 0.0004 | ***Bst2*** | 4.81 | 0.0004 |
| *Sprr1a* | + | 0.0004 | ***Ifit1*** | 5.84 | 0.0004 | ***Aoah*** | 4.81 | 0.0004 |
| *Lipg* | + | 0.0004 | ***Phf11d*** | 5.82 | 0.0004 | ***Ifi44*** | 4.81 | 0.0004 |
| *Ccl4* | + | 0.0004 | ***Ifi209*** | 5.68 | 0.0004 | ***Ptpn22*** | 4.79 | 0.0448 |
| *Ifit1bl1* | + | 0.0004 | ***Ifi206*** | 5.66 | 0.0442 | ***Ifit2*** | 4.79 | 0.0004 |
| *Acod1* | + | 0.0004 | ***Ifi213*** | 5.65 | 0.0004 | ***Cd40*** | 4.79 | 0.0004 |
| *Ly6c2* | 9.37 | 0.0004 | ***Cd300c2*** | 5.64 | 0.0344 | ***Iigp1*** | 4.77 | 0.0004 |
| *Cd300lf* | 8.72 | 0.0433 | ***Slfn8*** | 5.63 | 0.0004 | ***Ptafr*** | 4.75 | 0.0004 |
| *Mx1* | 8.39 | 0.0004 | ***Lair1*** | 5.61 | 0.0004 | ***H2-Q6*** | 4.74 | 0.0004 |
| *Ccl7* | 8.29 | 0.0011 | ***H2-Q7*** | 5.60 | 0.0004 | ***Pycard*** | 4.73 | 0.0004 |
| *Phf11b* | 8.16 | 0.0004 | ***Ms4a6d*** | 5.57 | 0.0004 | ***Rasgef1b*** | 4.72 | 0.0276 |
| *Ccl2* | 7.97 | 0.0004 | ***Lcn2*** | 5.56 | 0.0004 | ***Il2rg*** | 4.71 | 0.0004 |
| *Cxcl10* | 7.86 | 0.0004 | ***Gm13212*** | 5.55 | 0.0040 | ***Slfn2*** | 4.70 | 0.0004 |
| *Isg15* | 7.44 | 0.0004 | ***Sh2d6*** | 5.46 | 0.0004 | ***Pstpip1*** | 4.69 | 0.0004 |
| *Slc15a3* | 7.43 | 0.0023 | ***C3ar1*** | 5.45 | 0.0004 | ***Mcm5*** | 4.68 | 0.0004 |
| *Clec4e* | 7.35 | 0.0462 | ***Cd180*** | 5.40 | 0.0043 | ***Gbp2*** | 4.68 | 0.0004 |
| *Cxcl9* | 7.10 | 0.0174 | ***Dhx58*** | 5.35 | 0.0004 | ***Ms4a14*** | 4.66 | 0.0103 |
| *Chil1* | 6.86 | 0.0004 | ***Lpxn*** | 5.30 | 0.0004 | ***Xaf1*** | 4.63 | 0.0004 |
| *Arg1* | 6.69 | 0.0004 | ***Spp1*** | 5.29 | 0.0004 | ***Anxa8*** | 4.63 | 0.0004 |
| *Lockd* | 6.68 | 0.0004 | ***Oas2*** | 5.27 | 0.0004 | ***Tlr13*** | 4.63 | 0.0004 |
| *A530040E14Rik* | 6.62 | 0.0004 | ***Cd53*** | 5.26 | 0.0004 | ***Ms4a7*** | 4.62 | 0.0004 |
| *Slfn1* | 6.55 | 0.0004 | ***Klrk1*** | 5.25 | 0.0327 | ***Casp4*** | 4.62 | 0.0004 |
| *Ifit3* | 6.54 | 0.0004 | ***Cd52*** | 5.19 | 0.0004 | ***Gbp8*** | 4.62 | 0.0048 |
| *Oas3* | 6.48 | 0.0004 | ***Clec12a*** | 5.17 | 0.0004 | ***Cmpk2*** | 4.61 | 0.0004 |
| *Ccr5* | 6.46 | 0.0004 | ***Tlr7*** | 5.17 | 0.0004 | ***Ncf4*** | 4.61 | 0.0004 |
| *Zbp1* | 6.44 | 0.0004 | ***H2-T10*** | 5.12 | 0.0004 | ***Clec4a3*** | 4.60 | 0.0004 |
| *Ccl12* | 6.44 | 0.0004 | ***Cdc20*** | 5.09 | 0.0088 | ***Prr15*** | 4.60 | 0.0466 |
| *Timp1* | 6.39 | 0.0004 | ***Ly9*** | 5.07 | 0.0004 | ***Pirb*** | 4.59 | 0.0004 |
| *Fcgr1* | 6.36 | 0.0004 | ***Ctss*** | 5.07 | 0.0004 | ***Cd300ld*** | 4.59 | 0.0004 |
| *Plac8* | 6.35 | 0.0004 | ***Slfn9*** | 5.06 | 0.0330 | ***Batf2*** | 4.58 | 0.0060 |
| *Oas1a* | 6.32 | 0.0004 | ***Abcg1*** | 5.06 | 0.0004 | ***Trim30a*** | 4.57 | 0.0004 |
| *Lilr4b* | 6.31 | 0.0004 | ***Kif20b*** | 5.04 | 0.0004 | ***Tap1*** | 4.57 | 0.0004 |
| *Tgtp1* | 6.30 | 0.0281 | ***Lilrb4*** | 5.03 | 0.0004 | ***Cd68*** | 4.56 | 0.0004 |
| *Ccl5* | 6.25 | 0.0004 | ***Ifitm6*** | 5.03 | 0.0004 | ***Slc2a6*** | 4.55 | 0.0004 |
| *Tnfrsf11a* | 6.22 | 0.0004 | ***Apol9b*** | 5.01 | 0.0004 | ***Epsti1*** | 4.53 | 0.0004 |
| *Sell* | 6.22 | 0.0004 | ***Batf*** | 5.01 | 0.0004 | ***Ms4a4b*** | 4.51 | 0.0004 |

Table continuation:

| Gene  name | Log_2_ (FC) | *q*-value | Gene  name | Log_2_ (FC) | *q*-value | Gene  name | Log_2_ (FC) | *q*-value |
| --- | --- | --- | --- | --- | --- | --- | --- | --- |
| *Krt8* | 4.51 | 0.0004 | ***Il7r*** | 4.13 | 0.0017 | ***Foxm1*** | 3.85 | 0.0014 |
| *Ankrd1* | 4.49 | 0.0004 | ***Ifi30*** | 4.13 | 0.0004 | ***Cd5l*** | 3.84 | 0.0004 |
| *Fcer1g* | 4.48 | 0.0004 | ***Gbp3*** | 4.10 | 0.0004 | ***Glipr2*** | 3.84 | 0.0004 |
| *Dpep2* | 4.48 | 0.0243 | ***Rnase6*** | 4.10 | 0.0004 | ***Gm36161*** | 3.83 | 0.0329 |
| *Cdr2l* | 4.46 | 0.0004 | ***Krt18*** | 4.10 | 0.0004 | ***Ccl9*** | 3.83 | 0.0004 |
| *Ifit3b* | 4.46 | 0.0004 | ***Pclaf*** | 4.09 | 0.0348 | ***Il18bp*** | 3.82 | 0.0112 |
| *Ugt1a10* | 4.45 | 0.0481 | ***Rhoh*** | 4.09 | 0.0050 | ***Runx3*** | 3.82 | 0.0004 |
| *Mt1* | 4.44 | 0.0004 | ***H2-K1*** | 4.09 | 0.0004 | ***Themis2*** | 3.81 | 0.0004 |
| *Lacc1* | 4.44 | 0.0232 | ***Ube2l6*** | 4.09 | 0.0004 | ***Ly6a*** | 3.80 | 0.0004 |
| *Lst1* | 4.42 | 0.0004 | ***Fam180a*** | 4.08 | 0.0004 | ***Lcp1*** | 3.80 | 0.0004 |
| *Ccl8* | 4.41 | 0.0004 | ***Gpr35*** | 4.08 | 0.0004 | ***Aim2*** | 3.79 | 0.0040 |
| *Nlrc5* | 4.41 | 0.0004 | ***Trem2*** | 4.08 | 0.0004 | ***Tmsb10*** | 3.79 | 0.0004 |
| *Slc13a3* | 4.38 | 0.0004 | ***Trim30c*** | 4.07 | 0.0017 | ***Hpse*** | 3.78 | 0.0004 |
| *Slamf9* | 4.38 | 0.0004 | ***Ltb4r1*** | 4.07 | 0.0011 | ***Cfap45*** | 3.78 | 0.0011 |
| *Rtp4* | 4.36 | 0.0004 | ***Isg20*** | 4.06 | 0.0004 | ***Cdca8*** | 3.76 | 0.0035 |
| *Ripk3* | 4.36 | 0.0004 | ***Fcgr2b*** | 4.06 | 0.0004 | ***Sp110*** | 3.76 | 0.0004 |
| *Clec4a1* | 4.35 | 0.0004 | ***Lgals9*** | 4.06 | 0.0004 | ***Arpc1b*** | 3.76 | 0.0004 |
| *Pydc3* | 4.35 | 0.0404 | ***Evl*** | 4.05 | 0.0387 | ***Ifi27l2a*** | 3.73 | 0.0004 |
| *H2-Q4* | 4.33 | 0.0004 | ***Nfkbie*** | 4.05 | 0.0004 | ***Arl11*** | 3.73 | 0.0004 |
| *Lrrc25* | 4.31 | 0.0004 | ***Snx20*** | 4.04 | 0.0004 | ***Rgs14*** | 3.72 | 0.0004 |
| *Ifi205* | 4.30 | 0.0004 | ***Mmp3*** | 4.03 | 0.0004 | ***Gpr65*** | 3.72 | 0.0004 |
| *Mb21d1* | 4.29 | 0.0004 | ***H2-T22*** | 4.00 | 0.0004 | ***Mndal*** | 3.71 | 0.0004 |
| *Prps2* | 4.29 | 0.0004 | ***Trim30d*** | 4.00 | 0.0004 | ***Il10ra*** | 3.71 | 0.0004 |
| *Mefv* | 4.29 | 0.0004 | ***Marcksl1*** | 4.00 | 0.0004 | ***Cybb*** | 3.71 | 0.0004 |
| *Cfb* | 4.28 | 0.0004 | ***Mki67*** | 4.00 | 0.0004 | ***Myh3*** | 3.71 | 0.0004 |
| *Milr1* | 4.27 | 0.0008 | ***Pilra*** | 4.00 | 0.0004 | ***Mir703*** | 3.69 | 0.0441 |
| *H2-T23* | 4.27 | 0.0004 | ***Ikbke*** | 4.00 | 0.0004 | ***Parp12*** | 3.69 | 0.0004 |
| *Asns* | 4.27 | 0.0004 | ***Gm4951*** | 3.99 | 0.0004 | ***Ralgps2*** | 3.68 | 0.0101 |
| *Ccl6* | 4.26 | 0.0004 | ***Ifitm3*** | 3.99 | 0.0004 | ***Soat1*** | 3.68 | 0.0008 |
| *Mcemp1* | 4.26 | 0.0340 | ***Cd86*** | 3.99 | 0.0004 | ***Gm38316*** | 3.67 | 0.0217 |
| *Ctsw* | 4.25 | 0.0247 | ***Psmb9*** | 3.98 | 0.0004 | ***S100a4*** | 3.67 | 0.0004 |
| *Msr1* | 4.24 | 0.0004 | ***Tyms*** | 3.98 | 0.0004 | ***Aldh3b1*** | 3.67 | 0.0004 |
| *Alox5ap* | 4.24 | 0.0004 | ***Oas1c*** | 3.97 | 0.0497 | ***Mov10*** | 3.67 | 0.0004 |
| *Cd300lb* | 4.23 | 0.0014 | ***Misp*** | 3.97 | 0.0466 | ***Il13ra1*** | 3.66 | 0.0004 |
| *Gm34084* | 4.23 | 0.0011 | ***AI662270*** | 3.96 | 0.0004 | ***Itgb2*** | 3.65 | 0.0004 |
| *Rnf213* | 4.22 | 0.0004 | ***Cd44*** | 3.96 | 0.0004 | ***Ly86*** | 3.65 | 0.0004 |
| *Pik3r5* | 4.22 | 0.0004 | ***Hmox1*** | 3.96 | 0.0004 | ***Dlgap5*** | 3.65 | 0.0004 |
| *Pdpn* | 4.21 | 0.0004 | ***F10*** | 3.95 | 0.0004 | ***Gpnmb*** | 3.64 | 0.0004 |
| *Twist2* | 4.21 | 0.0008 | ***Tmem106a*** | 3.95 | 0.0004 | ***Gm26716*** | 3.64 | 0.0004 |
| *Gprc5a* | 4.20 | 0.0021 | ***Psat1*** | 3.93 | 0.0004 | ***Clec4a2*** | 3.63 | 0.0004 |
| *AC139671.1* | 4.19 | 0.0014 | ***Pnp*** | 3.93 | 0.0004 | ***Slc38a1*** | 3.62 | 0.0004 |
| *Oasl2* | 4.18 | 0.0004 | ***Psmb8*** | 3.92 | 0.0004 | ***Ada*** | 3.62 | 0.0261 |
| *Lcp2* | 4.18 | 0.0004 | ***Ifi203*** | 3.91 | 0.0004 | ***Krt7*** | 3.62 | 0.0004 |
| *Samsn1* | 4.18 | 0.0004 | ***Cd48*** | 3.91 | 0.0004 | ***Tyrobp*** | 3.61 | 0.0004 |
| *Ccr2* | 4.18 | 0.0004 | ***2010001K21Rik*** | 3.90 | 0.0178 | ***Plaur*** | 3.61 | 0.0004 |
| *Cxcl16* | 4.17 | 0.0004 | ***A630001G21Rik*** | 3.89 | 0.0004 | ***Ptgis*** | 3.60 | 0.0004 |
| *Ms4a8a* | 4.17 | 0.0004 | ***Galnt6*** | 3.88 | 0.0004 | ***Tor3a*** | 3.60 | 0.0004 |
| *Krt14* | 4.17 | 0.0004 | ***Slc23a4*** | 3.88 | 0.0004 | ***Gm2245*** | 3.60 | 0.0433 |
| *AB124611* | 4.16 | 0.0004 | ***Tesc*** | 3.88 | 0.0330 | ***Uhrf1*** | 3.59 | 0.0195 |
| *Casp1* | 4.16 | 0.0004 | ***Hmgb2*** | 3.87 | 0.0004 | ***Adar*** | 3.58 | 0.0004 |
| *Tmem51* | 4.15 | 0.0014 | ***Tifab*** | 3.87 | 0.0004 | ***Vav1*** | 3.58 | 0.0004 |
| *Slc6a4* | 4.14 | 0.0476 | ***Ckmt1*** | 3.86 | 0.0004 | ***Sp140*** | 3.58 | 0.0004 |
| *Dmkn* | 4.14 | 0.0004 | ***Pnp2*** | 3.86 | 0.0473 | ***Gmfg*** | 3.57 | 0.0344 |
| *BE692007* | 4.14 | 0.0004 | ***Apoc2*** | 3.86 | 0.0075 | ***Mcub*** | 3.57 | 0.0004 |
| *Top2a* | 4.13 | 0.0004 | ***Nckap1l*** | 3.86 | 0.0004 | ***Stat1*** | 3.57 | 0.0004 |

Table continuation:

| Gene  name | Log_2_ (FC) | *q*-value | Gene  name | Log_2_ (FC) | *q*-value | Gene  name | Log_2_ (FC) | *q*-value |
| --- | --- | --- | --- | --- | --- | --- | --- | --- |
| ***Metrnl*** | 3.57 | 0.0004 | ***Plk3*** | 3.39 | 0.0004 | ***Tuba1c*** | 3.16 | 0.0004 |
| ***Stambpl1*** | 3.57 | 0.0004 | ***Apobec1*** | 3.39 | 0.0004 | ***C1qa*** | 3.15 | 0.0004 |
| ***Myo1g*** | 3.57 | 0.0004 | ***Renbp*** | 3.39 | 0.0004 | ***Aplp1*** | 3.15 | 0.0004 |
| ***Plek*** | 3.56 | 0.0004 | ***Npas2*** | 3.39 | 0.0004 | ***Cfl1*** | 3.15 | 0.0004 |
| ***Muc16*** | 3.56 | 0.0004 | ***Rab32*** | 3.37 | 0.0004 | ***G530011O06Rik*** | 3.15 | 0.0004 |
| ***Edem1*** | 3.56 | 0.0004 | ***Avpr2*** | 3.37 | 0.0433 | ***Sdc3*** | 3.15 | 0.0004 |
| ***Igtp*** | 3.56 | 0.0004 | ***Cd274*** | 3.36 | 0.0004 | ***Acsl5*** | 3.15 | 0.0004 |
| ***Tlr2*** | 3.56 | 0.0004 | ***Smim3*** | 3.36 | 0.0004 | ***Gm5431*** | 3.15 | 0.0004 |
| ***H2-Q5*** | 3.55 | 0.0004 | ***Efhd2*** | 3.36 | 0.0004 | ***Pid1*** | 3.15 | 0.0004 |
| ***Herc6*** | 3.55 | 0.0035 | ***Helz2*** | 3.36 | 0.0004 | ***Ass1*** | 3.14 | 0.0004 |
| ***Slc37a2*** | 3.55 | 0.0004 | ***Krt19*** | 3.36 | 0.0004 | ***Adora2b*** | 3.14 | 0.0004 |
| ***Hpgd*** | 3.55 | 0.0004 | ***Samhd1*** | 3.36 | 0.0004 | ***Prim2*** | 3.14 | 0.0247 |
| ***Psd4*** | 3.54 | 0.0004 | ***Gm21188*** | 3.36 | 0.0004 | ***Slc7a4*** | 3.13 | 0.0143 |
| ***Zfp992*** | 3.54 | 0.0029 | ***Pdk3*** | 3.35 | 0.0017 | ***Tnfrsf14*** | 3.13 | 0.0004 |
| ***Fyb*** | 3.53 | 0.0004 | ***Serpinb6b*** | 3.34 | 0.0004 | ***Slc39a4*** | 3.13 | 0.0023 |
| ***Slc11a1*** | 3.52 | 0.0004 | ***Sfrp4*** | 3.33 | 0.0004 | ***Shisa5*** | 3.12 | 0.0004 |
| ***Csf3r*** | 3.52 | 0.0004 | ***Sp100*** | 3.33 | 0.0004 | ***Hps1*** | 3.12 | 0.0004 |
| ***Gch1*** | 3.52 | 0.0004 | ***Gbp5*** | 3.32 | 0.0004 | ***Upp1*** | 3.11 | 0.0004 |
| ***Coro1a*** | 3.52 | 0.0004 | ***Alcam*** | 3.32 | 0.0004 | ***Myh8*** | 3.11 | 0.0004 |
| ***Tnfrsf1b*** | 3.52 | 0.0004 | ***4933412E12Rik*** | 3.32 | 0.0254 | ***Samd9l*** | 3.10 | 0.0004 |
| ***Clic1*** | 3.52 | 0.0004 | ***Arhgdib*** | 3.31 | 0.0004 | ***Ppm1h*** | 3.10 | 0.0017 |
| ***Litaf*** | 3.51 | 0.0004 | ***Rap2b*** | 3.31 | 0.0004 | ***Nkain4*** | 3.09 | 0.0004 |
| ***Trim12a*** | 3.51 | 0.0004 | ***Reep4*** | 3.30 | 0.0417 | ***Emp3*** | 3.09 | 0.0004 |
| ***Ifi207*** | 3.51 | 0.0004 | ***Tmem151a*** | 3.29 | 0.0004 | ***Plk1*** | 3.08 | 0.0029 |
| ***Siglec1*** | 3.51 | 0.0004 | ***Il18*** | 3.29 | 0.0008 | ***Plscr1*** | 3.08 | 0.0008 |
| ***Wfdc17*** | 3.50 | 0.0004 | ***Bex3*** | 3.28 | 0.0004 | ***Srgn*** | 3.07 | 0.0004 |
| ***Tpcn2*** | 3.50 | 0.0004 | ***Rexo5*** | 3.28 | 0.0167 | ***Anxa2*** | 3.07 | 0.0004 |
| ***Ptpn6*** | 3.49 | 0.0004 | ***Uba7*** | 3.27 | 0.0004 | ***Dok1*** | 3.06 | 0.0004 |
| ***Hcls1*** | 3.49 | 0.0004 | ***Myc*** | 3.27 | 0.0004 | ***Fcgr3*** | 3.06 | 0.0004 |
| ***Tapbp*** | 3.49 | 0.0004 | ***H2-M3*** | 3.27 | 0.0004 | ***Pafah1b3*** | 3.05 | 0.0004 |
| ***Unc93b1*** | 3.49 | 0.0004 | ***Akr1b8*** | 3.27 | 0.0004 | ***Tubb2b*** | 3.05 | 0.0004 |
| ***Oas1b*** | 3.49 | 0.0004 | ***C5ar1*** | 3.27 | 0.0004 | ***Traf1*** | 3.05 | 0.0004 |
| ***Grn*** | 3.49 | 0.0004 | ***Slc39a8*** | 3.26 | 0.0004 | ***Slc9a3r1*** | 3.05 | 0.0004 |
| ***Arhgap33*** | 3.49 | 0.0476 | ***Cyth4*** | 3.25 | 0.0004 | ***Evi2a*** | 3.05 | 0.0004 |
| ***Stmn1*** | 3.49 | 0.0004 | ***Ncf2*** | 3.25 | 0.0004 | ***Tsku*** | 3.03 | 0.0004 |
| ***Spi1*** | 3.48 | 0.0004 | ***Nfam1*** | 3.25 | 0.0004 | ***Capg*** | 3.03 | 0.0004 |
| ***Serpine1*** | 3.48 | 0.0008 | ***Vcan*** | 3.24 | 0.0053 | ***Cdk6*** | 3.02 | 0.0060 |
| ***Kcnn4*** | 3.47 | 0.0017 | ***Npc2*** | 3.24 | 0.0004 | ***Apbb1ip*** | 3.02 | 0.0004 |
| ***Lgals3bp*** | 3.47 | 0.0004 | ***Aldh1a2*** | 3.23 | 0.0004 | ***P2ry14*** | 3.02 | 0.0067 |
| ***Actb*** | 3.46 | 0.0004 | ***Vim*** | 3.23 | 0.0004 | ***Kng2*** | 3.02 | 0.0004 |
| ***H2-D1*** | 3.46 | 0.0004 | ***Myl4*** | 3.23 | 0.0004 | ***Nupr1*** | 3.02 | 0.0004 |
| ***Mgp*** | 3.46 | 0.0004 | ***Apod*** | 3.22 | 0.0004 | ***Lamc3*** | 3.01 | 0.0004 |
| ***Msln*** | 3.44 | 0.0004 | ***Gm8995*** | 3.21 | 0.0004 | ***Pck2*** | 3.01 | 0.0004 |
| ***Bcl3*** | 3.44 | 0.0004 | ***Arhgap4*** | 3.21 | 0.0037 | ***Kif23*** | 3.01 | 0.0008 |
| ***Il1rl1*** | 3.44 | 0.0040 | ***Gsdmd*** | 3.21 | 0.0004 | ***Cfap43*** | 3.00 | 0.0099 |
| ***Ncf1*** | 3.42 | 0.0004 | ***Cela1*** | 3.20 | 0.0004 | ***Irgm2*** | 3.00 | 0.0004 |
| ***F7*** | 3.42 | 0.0037 | ***Chrnb2*** | 3.19 | 0.0004 | ***Rad51ap1*** | 3.00 | 0.0055 |
| ***Ctsk*** | 3.42 | 0.0004 | ***Myof*** | 3.19 | 0.0004 | ***Efna5*** | 2.99 | 0.0004 |
| ***B2m*** | 3.41 | 0.0004 | ***Gm2564*** | 3.18 | 0.0004 | ***Lyn*** | 2.99 | 0.0004 |
| ***Cstb*** | 3.41 | 0.0004 | ***Parp14*** | 3.18 | 0.0004 | ***Fam111a*** | 2.99 | 0.0004 |
| ***Irf5*** | 3.41 | 0.0497 | ***Gm47079*** | 3.18 | 0.0008 | ***Nrcam*** | 2.98 | 0.0004 |
| ***Basp1*** | 3.41 | 0.0004 | ***Pou2f2*** | 3.18 | 0.0004 | ***Mmp14*** | 2.97 | 0.0004 |
| ***Pik3ap1*** | 3.40 | 0.0023 | ***Serpinb1a*** | 3.18 | 0.0004 | ***Zbtb7c*** | 2.97 | 0.0004 |
| ***Il18r1*** | 3.40 | 0.0004 | ***Bub1b*** | 3.16 | 0.0014 | ***Tcirg1*** | 2.96 | 0.0004 |
| ***Ly6e*** | 3.40 | 0.0004 | ***Adgre1*** | 3.16 | 0.0004 | ***C130026I21Rik*** | 2.96 | 0.0075 |

Table continuation:

| Gene  name | Log_2_ (FC) | *q*-value | Gene  name | Log_2_ (FC) | *q*-value | Gene  name | Log_2_ (FC) | *q*-value |
| --- | --- | --- | --- | --- | --- | --- | --- | --- |
| ***Kcnab2*** | 2.96 | 0.0004 | ***Gbp4*** | 2.79 | 0.0004 | ***Arhgap25*** | 2.69 | 0.0026 |
| ***C1qc*** | 2.96 | 0.0004 | ***Tcf19*** | 2.79 | 0.0004 | ***Naip2*** | 2.69 | 0.0004 |
| ***C1qb*** | 2.96 | 0.0004 | ***Plxnb2*** | 2.79 | 0.0004 | ***Mitd1*** | 2.68 | 0.0004 |
| ***Lypd1*** | 2.95 | 0.0116 | ***Tfpi*** | 2.79 | 0.0004 | ***Rbm43*** | 2.68 | 0.0004 |
| ***Sirpa*** | 2.95 | 0.0004 | ***Fermt3*** | 2.78 | 0.0079 | ***Fbxw17*** | 2.68 | 0.0004 |
| ***Nfe2*** | 2.95 | 0.0004 | ***Postn*** | 2.78 | 0.0004 | ***Vnn1*** | 2.68 | 0.0004 |
| ***Ptpn7*** | 2.95 | 0.0004 | ***Dlg4*** | 2.78 | 0.0004 | ***Csrp2*** | 2.67 | 0.0004 |
| ***Ahnak2*** | 2.94 | 0.0004 | ***Tubb5*** | 2.78 | 0.0004 | ***Slc22a18*** | 2.67 | 0.0004 |
| ***Fabp5*** | 2.94 | 0.0004 | ***Scpep1*** | 2.78 | 0.0004 | ***Mfsd7a*** | 2.67 | 0.0008 |
| ***Gjb5*** | 2.94 | 0.0004 | ***Gbp7*** | 2.78 | 0.0004 | ***Socs1*** | 2.67 | 0.0004 |
| ***Selplg*** | 2.94 | 0.0004 | ***Olfr1033*** | 2.78 | 0.0004 | ***Itgb7*** | 2.67 | 0.0004 |
| ***Frrs1*** | 2.93 | 0.0004 | ***Mt3*** | 2.77 | 0.0004 | ***Gm45551*** | 2.67 | 0.0004 |
| ***Faah*** | 2.93 | 0.0011 | ***Abhd2*** | 2.77 | 0.0004 | ***Spib*** | 2.67 | 0.0004 |
| ***Plekho2*** | 2.93 | 0.0004 | ***Lrrc1*** | 2.77 | 0.0004 | ***Ncam1*** | 2.66 | 0.0008 |
| ***Dock2*** | 2.92 | 0.0004 | ***Ftl1*** | 2.77 | 0.0004 | ***Enkd1*** | 2.66 | 0.0385 |
| ***Mapk13*** | 2.92 | 0.0004 | ***Gpr137b-ps*** | 2.76 | 0.0004 | ***Layn*** | 2.66 | 0.0004 |
| ***Ms4a4d*** | 2.92 | 0.0004 | ***Arhgap30*** | 2.76 | 0.0004 | ***Adam8*** | 2.65 | 0.0011 |
| ***Mmp27*** | 2.92 | 0.0058 | ***C1ra*** | 2.76 | 0.0004 | ***Cdk14*** | 2.65 | 0.0004 |
| ***Sash3*** | 2.92 | 0.0004 | ***Dram1*** | 2.76 | 0.0004 | ***S100a10*** | 2.65 | 0.0004 |
| ***Tagln2*** | 2.91 | 0.0004 | ***Dok2*** | 2.76 | 0.0008 | ***Slc7a1*** | 2.65 | 0.0004 |
| ***Sptlc2*** | 2.91 | 0.0014 | ***Lbh*** | 2.76 | 0.0004 | ***Nipsnap3b*** | 2.64 | 0.0029 |
| ***Gbp6*** | 2.91 | 0.0004 | ***1700017B05Rik*** | 2.75 | 0.0004 | ***Zcchc3*** | 2.64 | 0.0004 |
| ***Plcg2*** | 2.91 | 0.0004 | ***Mfsd12*** | 2.75 | 0.0004 | ***Mfsd10*** | 2.64 | 0.0004 |
| ***Fap*** | 2.90 | 0.0004 | ***Ucp2*** | 2.75 | 0.0004 | ***Kcnd1*** | 2.64 | 0.0004 |
| ***Phgdh*** | 2.90 | 0.0004 | ***Ttc39b*** | 2.74 | 0.0088 | ***Rab31*** | 2.64 | 0.0004 |
| ***Tnfaip8l2*** | 2.89 | 0.0004 | ***Sat1*** | 2.74 | 0.0004 | ***Agpat4*** | 2.64 | 0.0004 |
| ***Rab7b*** | 2.89 | 0.0004 | ***Adamtsl1*** | 2.74 | 0.0004 | ***Ccdc88b*** | 2.63 | 0.0004 |
| ***Gm47761*** | 2.89 | 0.0004 | ***Was*** | 2.74 | 0.0110 | ***Mirt1*** | 2.63 | 0.0004 |
| ***Laptm5*** | 2.89 | 0.0004 | ***Irf1*** | 2.74 | 0.0004 | ***Fam46a*** | 2.62 | 0.0004 |
| ***Nrros*** | 2.88 | 0.0004 | ***Tmem176b*** | 2.74 | 0.0004 | ***Cd14*** | 2.62 | 0.0004 |
| ***Ptk2b*** | 2.88 | 0.0004 | ***Cotl1*** | 2.74 | 0.0004 | ***Ppp1r18*** | 2.61 | 0.0004 |
| ***Gm10684*** | 2.88 | 0.0466 | ***Clec5a*** | 2.73 | 0.0011 | ***Ifi203-ps*** | 2.61 | 0.0004 |
| ***Gm14005*** | 2.87 | 0.0004 | ***P2rx7*** | 2.73 | 0.0004 | ***Pdzrn4*** | 2.61 | 0.0026 |
| ***Napsa*** | 2.87 | 0.0004 | ***Parp11*** | 2.73 | 0.0004 | ***Sting*** | 2.60 | 0.0004 |
| ***Ly96*** | 2.87 | 0.0004 | ***Coro2a*** | 2.73 | 0.0330 | ***Dennd2d*** | 2.60 | 0.0491 |
| ***Emb*** | 2.86 | 0.0004 | ***H2-DMb1*** | 2.73 | 0.0004 | ***Tap2*** | 2.60 | 0.0339 |
| ***Cap1*** | 2.85 | 0.0004 | ***Lig1*** | 2.73 | 0.0178 | ***Gna15*** | 2.60 | 0.0004 |
| ***AI467606*** | 2.85 | 0.0004 | ***Lipa*** | 2.73 | 0.0004 | ***Mrgprf*** | 2.60 | 0.0004 |
| ***Pld4*** | 2.84 | 0.0004 | ***Vat1*** | 2.73 | 0.0004 | ***Slc9a9*** | 2.60 | 0.0004 |
| ***Fkbp1b*** | 2.84 | 0.0065 | ***Mst1r*** | 2.72 | 0.0004 | ***Xdh*** | 2.60 | 0.0004 |
| ***Gm9844*** | 2.84 | 0.0070 | ***Car13*** | 2.72 | 0.0004 | ***Vill*** | 2.60 | 0.0004 |
| ***Ifih1*** | 2.84 | 0.0004 | ***Gm12840*** | 2.72 | 0.0004 | ***Shtn1*** | 2.59 | 0.0004 |
| ***Sugct*** | 2.83 | 0.0008 | ***Bmper*** | 2.72 | 0.0004 | ***Nrm*** | 2.59 | 0.0424 |
| ***Csf2ra*** | 2.83 | 0.0004 | ***Gm17711*** | 2.72 | 0.0004 | ***Cyp4v3*** | 2.59 | 0.0004 |
| ***Pilrb1*** | 2.82 | 0.0004 | ***Pkib*** | 2.72 | 0.0090 | ***Col1a1*** | 2.59 | 0.0004 |
| ***Il3ra*** | 2.82 | 0.0004 | ***Myog*** | 2.72 | 0.0004 | ***Stard5*** | 2.59 | 0.0070 |
| ***Rac2*** | 2.82 | 0.0014 | ***Cyp2s1*** | 2.71 | 0.0004 | ***Arpc5*** | 2.59 | 0.0004 |
| ***Bcat1*** | 2.82 | 0.0029 | ***Prkcb*** | 2.71 | 0.0004 | ***Neurl3*** | 2.58 | 0.0004 |
| ***Hpgds*** | 2.82 | 0.0011 | ***Irgm1*** | 2.71 | 0.0004 | ***C2*** | 2.58 | 0.0045 |
| ***Fxyd5*** | 2.82 | 0.0004 | ***Gm4841*** | 2.71 | 0.0004 | ***Cd33*** | 2.58 | 0.0070 |
| ***Cdh3*** | 2.81 | 0.0004 | ***Cmtm3*** | 2.71 | 0.0004 | ***Prg4*** | 2.57 | 0.0004 |
| ***Upk1b*** | 2.81 | 0.0004 | ***Wisp2*** | 2.70 | 0.0004 | ***Lgmn*** | 2.57 | 0.0004 |
| ***Dapp1*** | 2.81 | 0.0004 | ***Cklf*** | 2.69 | 0.0118 | ***Psd*** | 2.57 | 0.0004 |
| ***Arhgap9*** | 2.81 | 0.0037 | ***Plcb2*** | 2.69 | 0.0026 | ***Anpep*** | 2.57 | 0.0004 |
| ***Efs*** | 2.81 | 0.0004 | ***Stat2*** | 2.69 | 0.0004 | ***Cfp*** | 2.57 | 0.0004 |

Table continuation:

| Gene  name | Log_2_ (FC) | *q*-value | Gene  name | Log_2_ (FC) | *q*-value | Gene  name | Log_2_ (FC) | *q*-value |
| --- | --- | --- | --- | --- | --- | --- | --- | --- |
| *Guca1a* | 2.57 | 0.0004 | ***Nfe2l3*** | 2.47 | 0.0014 | ***S100a11*** | 2.35 | 0.0004 |
| *Ifnar2* | 2.57 | 0.0004 | ***Ubash3b*** | 2.46 | 0.0157 | ***Adamts14*** | 2.35 | 0.0004 |
| *Mcm6* | 2.57 | 0.0004 | ***Tmsb4x*** | 2.46 | 0.0004 | ***Tenm4*** | 2.35 | 0.0004 |
| *Tmem221* | 2.57 | 0.0090 | ***Itgam*** | 2.46 | 0.0004 | ***Cd83*** | 2.35 | 0.0011 |
| *Folr2* | 2.56 | 0.0004 | ***Il4ra*** | 2.46 | 0.0004 | ***Tyro3*** | 2.35 | 0.0004 |
| *Ptpn18* | 2.56 | 0.0004 | ***Styk1*** | 2.46 | 0.0293 | ***Tbc1d10a*** | 2.35 | 0.0008 |
| *Serpinb8* | 2.56 | 0.0491 | ***Arhgap45*** | 2.46 | 0.0004 | ***Rtn1*** | 2.35 | 0.0004 |
| *Pde1b* | 2.56 | 0.0004 | ***Ptgir*** | 2.46 | 0.0004 | ***S100a6*** | 2.35 | 0.0004 |
| *Serpinb9* | 2.56 | 0.0004 | ***Adora1*** | 2.45 | 0.0004 | ***Mob3a*** | 2.35 | 0.0004 |
| *Eif2ak2* | 2.56 | 0.0004 | ***Ptprc*** | 2.45 | 0.0008 | ***Prr13*** | 2.34 | 0.0004 |
| *Ctsh* | 2.55 | 0.0004 | ***Cpxm1*** | 2.45 | 0.0004 | ***Pitx1*** | 2.34 | 0.0004 |
| *Tmem229b* | 2.55 | 0.0023 | ***Ccr1*** | 2.44 | 0.0004 | ***Exoc3l4*** | 2.33 | 0.0017 |
| *Sh3pxd2b* | 2.55 | 0.0004 | ***Daxx*** | 2.44 | 0.0004 | ***Dact3*** | 2.33 | 0.0004 |
| *Plk2* | 2.55 | 0.0004 | ***Mcm4*** | 2.44 | 0.0334 | ***P2rx4*** | 2.33 | 0.0004 |
| *Ptgs1* | 2.55 | 0.0004 | ***Nxnl2*** | 2.44 | 0.0123 | ***Cldn15*** | 2.33 | 0.0004 |
| *Myrf* | 2.54 | 0.0004 | ***Tmc6*** | 2.44 | 0.0004 | ***Cdca3*** | 2.33 | 0.0212 |
| *Myo1f* | 2.54 | 0.0014 | ***Itgb3*** | 2.44 | 0.0004 | ***Tapbpl*** | 2.33 | 0.0004 |
| *Tmem86a* | 2.54 | 0.0004 | ***Gsap*** | 2.43 | 0.0357 | ***Pgm1*** | 2.33 | 0.0079 |
| *AW112010* | 2.54 | 0.0004 | ***Susd3*** | 2.43 | 0.0008 | ***Adap1*** | 2.32 | 0.0004 |
| *Gm26809* | 2.53 | 0.0004 | ***Arf6*** | 2.43 | 0.0004 | ***Eps8*** | 2.32 | 0.0103 |
| *Timd4* | 2.53 | 0.0011 | ***Wwc1*** | 2.43 | 0.0023 | ***Arap1*** | 2.32 | 0.0004 |
| *Tmem98* | 2.53 | 0.0004 | ***Cenpa*** | 2.43 | 0.0014 | ***Tubb2a*** | 2.32 | 0.0004 |
| *Cd37* | 2.53 | 0.0004 | ***Osbpl3*** | 2.43 | 0.0004 | ***2810474O19Rik*** | 2.32 | 0.0004 |
| *Ifitm2* | 2.53 | 0.0004 | ***Prim1*** | 2.43 | 0.0026 | ***Coro1c*** | 2.32 | 0.0004 |
| *1500011B03Rik* | 2.53 | 0.0017 | ***Prc1*** | 2.43 | 0.0004 | ***Eif4a1*** | 2.32 | 0.0004 |
| *Ncaph* | 2.53 | 0.0026 | ***Zc3hav1*** | 2.42 | 0.0004 | ***Mxra8*** | 2.32 | 0.0004 |
| *Atp1a3* | 2.53 | 0.0004 | ***Anxa1*** | 2.42 | 0.0004 | ***Nradd*** | 2.32 | 0.0004 |
| *Sytl1* | 2.52 | 0.0004 | ***Igha*** | 2.42 | 0.0004 | ***Psme2*** | 2.32 | 0.0004 |
| *Fetub* | 2.52 | 0.0004 | ***Carhsp1*** | 2.42 | 0.0004 | ***Irak4*** | 2.31 | 0.0385 |
| *Ccnd1* | 2.52 | 0.0004 | ***Elf4*** | 2.42 | 0.0004 | ***Osmr*** | 2.31 | 0.0060 |
| *Trim34a* | 2.52 | 0.0035 | ***Rasal3*** | 2.42 | 0.0095 | ***Tgfb1*** | 2.31 | 0.0004 |
| *Ces2g* | 2.51 | 0.0004 | ***Sh3bp1*** | 2.41 | 0.0123 | ***Sri*** | 2.31 | 0.0004 |
| *Hsh2d* | 2.51 | 0.0017 | ***Ddx58*** | 2.41 | 0.0032 | ***Emp1*** | 2.30 | 0.0004 |
| *Slc3a2* | 2.51 | 0.0004 | ***Dpysl2*** | 2.41 | 0.0004 | ***Ftl1-ps1*** | 2.30 | 0.0004 |
| *Fblim1* | 2.51 | 0.0004 | ***Itgax*** | 2.41 | 0.0055 | ***B3gnt7*** | 2.30 | 0.0083 |
| *Lyz2* | 2.51 | 0.0004 | ***Tm4sf1*** | 2.41 | 0.0004 | ***Cercam*** | 2.30 | 0.0004 |
| *Slc1a4* | 2.51 | 0.0004 | ***Cdc42se1*** | 2.41 | 0.0093 | ***Cnmd*** | 2.30 | 0.0035 |
| *Tbc1d10c* | 2.50 | 0.0026 | ***BC028528*** | 2.41 | 0.0004 | ***Fam105a*** | 2.30 | 0.0467 |
| *Stc2* | 2.50 | 0.0035 | ***Csf2rb*** | 2.41 | 0.0004 | ***Ralb*** | 2.30 | 0.0004 |
| *Larp6* | 2.50 | 0.0035 | ***Racgap1*** | 2.40 | 0.0004 | ***Trim21*** | 2.30 | 0.0239 |
| *Gm9973* | 2.49 | 0.0004 | ***Casp8*** | 2.40 | 0.0004 | ***Cxcr3*** | 2.30 | 0.0187 |
| *Mir142hg* | 2.49 | 0.0004 | ***Cpt1c*** | 2.39 | 0.0004 | ***Cldn10*** | 2.30 | 0.0004 |
| *Gda* | 2.49 | 0.0004 | ***AC121567.1*** | 2.39 | 0.0004 | ***Dok3*** | 2.30 | 0.0004 |
| *Irf9* | 2.49 | 0.0004 | ***Tor4a*** | 2.39 | 0.0004 | ***Sbsn*** | 2.30 | 0.0004 |
| *Pik3r6* | 2.49 | 0.0008 | ***Inpp5d*** | 2.39 | 0.0004 | ***Fads3*** | 2.29 | 0.0004 |
| *Ptprj* | 2.49 | 0.0004 | ***Tmem176a*** | 2.38 | 0.0004 | ***Btg1*** | 2.29 | 0.0004 |
| *Gata5* | 2.48 | 0.0153 | ***Lgals2*** | 2.38 | 0.0112 | ***Itgal*** | 2.28 | 0.0106 |
| *Dclk1* | 2.48 | 0.0004 | ***Plau*** | 2.38 | 0.0004 | ***Naglu*** | 2.28 | 0.0004 |
| *9930111J21Rik2* | 2.48 | 0.0004 | ***Jpt2*** | 2.38 | 0.0004 | ***Fkbp10*** | 2.28 | 0.0004 |
| *Sesn2* | 2.48 | 0.0004 | ***Sv2a*** | 2.37 | 0.0400 | ***Fstl1*** | 2.28 | 0.0004 |
| *Trim25* | 2.47 | 0.0004 | ***Smc4*** | 2.37 | 0.0004 | ***Tax1bp3*** | 2.27 | 0.0004 |
| *Gm37401* | 2.47 | 0.0004 | ***Sh3tc2*** | 2.36 | 0.0004 | ***Ctsc*** | 2.27 | 0.0004 |
| *C4b* | 2.47 | 0.0004 | ***Adamts15*** | 2.36 | 0.0004 | ***Col4a6*** | 2.27 | 0.0004 |
| *Id2* | 2.47 | 0.0004 | ***Isyna1*** | 2.36 | 0.0004 | ***Cacnb3*** | 2.27 | 0.0004 |
| *Spon1* | 2.47 | 0.0004 | ***Sdsl*** | 2.35 | 0.0014 | ***C1rl*** | 2.26 | 0.0004 |

Table continuation:

| Gene  name | Log_2_ (FC) | *q*-value | Gene  name | Log_2_ (FC) | *q*-value | Gene  name | Log_2_ (FC) | *q*-value |
| --- | --- | --- | --- | --- | --- | --- | --- | --- |
| *Gm15512* | 2.26 | 0.0004 | ***Uap1l1*** | 2.17 | 0.0004 | ***Alkbh2*** | 2.09 | 0.0258 |
| *Enc1* | 2.26 | 0.0004 | ***Abcc3*** | 2.17 | 0.0081 | ***Cebpd*** | 2.09 | 0.0004 |
| *Faap24* | 2.26 | 0.0063 | ***Tmem14a*** | 2.17 | 0.0075 | ***Ltc4s*** | 2.09 | 0.0004 |
| *Spata6* | 2.26 | 0.0004 | ***Fes*** | 2.16 | 0.0004 | ***AI661453*** | 2.09 | 0.0011 |
| *Padi4* | 2.25 | 0.0070 | ***Hhex*** | 2.16 | 0.0081 | ***Rhoc*** | 2.08 | 0.0004 |
| *Hp* | 2.25 | 0.0004 | ***Fbln7*** | 2.16 | 0.0004 | ***Mdfic*** | 2.08 | 0.0004 |
| *Hhipl1* | 2.25 | 0.0035 | ***Gm17134*** | 2.16 | 0.0004 | ***Prdx4*** | 2.08 | 0.0004 |
| *Bin3* | 2.25 | 0.0004 | ***Pdia3*** | 2.16 | 0.0004 | ***Plat*** | 2.08 | 0.0004 |
| *Slc7a7* | 2.25 | 0.0048 | ***Crtap*** | 2.16 | 0.0004 | ***Lfng*** | 2.08 | 0.0004 |
| *Rgs10* | 2.25 | 0.0004 | ***Tmem198b*** | 2.16 | 0.0125 | ***Parva*** | 2.08 | 0.0004 |
| *Gas7* | 2.24 | 0.0032 | ***Gla*** | 2.16 | 0.0149 | ***Gm26527*** | 2.08 | 0.0342 |
| *Srd5a3* | 2.24 | 0.0004 | ***Tmem119*** | 2.16 | 0.0004 | ***Spint2*** | 2.07 | 0.0004 |
| *Nfkbiz* | 2.24 | 0.0004 | ***Wfdc21*** | 2.15 | 0.0004 | ***D6Wsu163e*** | 2.07 | 0.0032 |
| *Cpt1a* | 2.24 | 0.0004 | ***Psmb10*** | 2.15 | 0.0004 | ***Serpinh1*** | 2.07 | 0.0004 |
| *Batf3* | 2.24 | 0.0004 | ***Arhgap28*** | 2.15 | 0.0139 | ***Rassf7*** | 2.07 | 0.0004 |
| *Gnb1* | 2.23 | 0.0004 | ***Pglyrp1*** | 2.15 | 0.0014 | ***Clec10a*** | 2.06 | 0.0004 |
| *Tlr3* | 2.23 | 0.0129 | ***Thbs4*** | 2.14 | 0.0004 | ***BC017643*** | 2.06 | 0.0023 |
| *Sdf2l1* | 2.23 | 0.0004 | ***Gpr132*** | 2.14 | 0.0048 | ***Snhg12*** | 2.06 | 0.0011 |
| *Dock11* | 2.22 | 0.0004 | ***Stard3nl*** | 2.14 | 0.0004 | ***Agrn*** | 2.06 | 0.0004 |
| *S1pr2* | 2.22 | 0.0004 | ***Glb1*** | 2.14 | 0.0014 | ***Cyp4f18*** | 2.06 | 0.0133 |
| *Faim* | 2.22 | 0.0023 | ***Slc28a2*** | 2.14 | 0.0017 | ***Sgpl1*** | 2.05 | 0.0004 |
| *AI429214* | 2.22 | 0.0095 | ***Anxa5*** | 2.14 | 0.0004 | ***2410006H16Rik*** | 2.05 | 0.0004 |
| *Sod3* | 2.21 | 0.0004 | ***Cd209f*** | 2.13 | 0.0004 | ***Smim24*** | 2.05 | 0.0004 |
| *Slc10a6* | 2.21 | 0.0014 | ***Fam92a*** | 2.13 | 0.0004 | ***Elovl1*** | 2.05 | 0.0004 |
| *Pgam1* | 2.21 | 0.0004 | ***Cd47*** | 2.13 | 0.0004 | ***Clcn5*** | 2.04 | 0.0004 |
| *Plekha4* | 2.21 | 0.0004 | ***Ninl*** | 2.13 | 0.0004 | ***Sdc4*** | 2.04 | 0.0004 |
| *Atp10a* | 2.21 | 0.0004 | ***Commd10*** | 2.13 | 0.0004 | ***Iqgap2*** | 2.04 | 0.0004 |
| *Actn1* | 2.21 | 0.0004 | ***Ctps2*** | 2.13 | 0.0004 | ***Rgs19*** | 2.04 | 0.0004 |
| *Ckap4* | 2.20 | 0.0004 | ***Pml*** | 2.13 | 0.0004 | ***Zfp995*** | 2.04 | 0.0286 |
| *Igfbp3* | 2.20 | 0.0004 | ***Irak2*** | 2.13 | 0.0004 | ***Gucy1a1*** | 2.04 | 0.0026 |
| *Kdelr2* | 2.20 | 0.0004 | ***Gpr137b*** | 2.13 | 0.0004 | ***Gdf10*** | 2.04 | 0.0008 |
| *Baiap2* | 2.20 | 0.0004 | ***Csk*** | 2.13 | 0.0004 | ***Ptpn13*** | 2.04 | 0.0004 |
| *Syk* | 2.20 | 0.0004 | ***Tspo*** | 2.12 | 0.0004 | ***Cbr3*** | 2.04 | 0.0008 |
| *Ppib* | 2.20 | 0.0035 | ***Lbp*** | 2.12 | 0.0004 | ***Eef1a1*** | 2.04 | 0.0004 |
| *Ankrd13d* | 2.20 | 0.0067 | ***Cdkn1a*** | 2.12 | 0.0004 | ***Cyp4f16*** | 2.03 | 0.0008 |
| *Snx1* | 2.19 | 0.0004 | ***Cep128*** | 2.12 | 0.0004 | ***B4galt6*** | 2.03 | 0.0004 |
| *Baz1a* | 2.19 | 0.0271 | ***Sema4b*** | 2.12 | 0.0077 | ***Scnn1a*** | 2.03 | 0.0058 |
| *2610016A17Rik* | 2.19 | 0.0037 | ***Acer3*** | 2.12 | 0.0413 | ***G6pdx*** | 2.03 | 0.0023 |
| *Ppp1r9a* | 2.19 | 0.0163 | ***Ctsb*** | 2.11 | 0.0004 | ***Acap1*** | 2.02 | 0.0023 |
| *Edem2* | 2.19 | 0.0004 | ***Hpcal1*** | 2.11 | 0.0004 | ***Adcyap1r1*** | 2.02 | 0.0004 |
| *Faap20* | 2.18 | 0.0004 | ***Actc1*** | 2.11 | 0.0004 | ***Eno1*** | 2.02 | 0.0004 |
| *Coro1b* | 2.18 | 0.0004 | ***Trim5*** | 2.11 | 0.0004 | ***Spg21*** | 2.02 | 0.0004 |
| *Bicdl1* | 2.18 | 0.0004 | ***Sla*** | 2.11 | 0.0014 | ***Ngf*** | 2.02 | 0.0021 |
| *Adap2* | 2.18 | 0.0004 | ***Mmp23*** | 2.11 | 0.0004 | ***Nuak2*** | 2.02 | 0.0040 |
| *Fmnl1* | 2.18 | 0.0004 | ***Tuba1b*** | 2.11 | 0.0004 | ***Sh3bgrl3*** | 2.01 | 0.0004 |
| *Dtx3l* | 2.18 | 0.0004 | ***Twf1*** | 2.10 | 0.0004 | ***Nmi*** | 2.01 | 0.0004 |
| *Map4k1* | 2.18 | 0.0029 | ***Nxpe5*** | 2.10 | 0.0004 | ***Adgrg2*** | 2.01 | 0.0149 |
| *Egr1* | 2.18 | 0.0004 | ***Ywhaz*** | 2.10 | 0.0004 | ***Itpr3*** | 2.01 | 0.0004 |
| *Arl4c* | 2.18 | 0.0004 | ***B4galt5*** | 2.10 | 0.0075 | ***Pnmt*** | 2.01 | 0.0004 |
| *Arrb2* | 2.17 | 0.0004 | ***Tspan4*** | 2.10 | 0.0008 | ***Cgn*** | 2.01 | 0.0004 |
| *Lrmp* | 2.17 | 0.0376 | ***Amigo2*** | 2.10 | 0.0053 | ***Klf6*** | 2.01 | 0.0004 |
| *Vnn3* | 2.17 | 0.0327 | ***Mapk7*** | 2.10 | 0.0223 | ***Gfpt2*** | 2.01 | 0.0004 |
| *Rgl1* | 2.17 | 0.0004 | ***Il10rb*** | 2.10 | 0.0131 | ***Aif1l*** | 2.01 | 0.0004 |
| *Pdia5* | 2.17 | 0.0004 | ***Ptma*** | 2.09 | 0.0004 | ***Incenp*** | 2.00 | 0.0004 |
| *Orai2* | 2.17 | 0.0004 | ***Nipsnap1*** | 2.09 | 0.0008 |  |  |  |

Table continuation:

| Gene  name | Log_2_ (FC) | *q*-value | Gene name | Log_2_ (FC) | *q*-value | Gene  name | Log_2_ (FC) | *q*-value |
| --- | --- | --- | --- | --- | --- | --- | --- | --- |
| *Gm22993* | −2.01 | 0.0045 | ***Lars2*** | −2.24 | 0.0004 | ***Kctd7*** | −2.72 | 0.0141 |
| *Dpf1* | −2.01 | 0.0225 | ***Gck*** | −2.25 | 0.0004 | ***Slc25a30*** | −2.73 | 0.0145 |
| *Gm10643* | −2.02 | 0.0029 | ***March8*** | −2.28 | 0.0291 | ***Zbtb18*** | −2.74 | 0.0058 |
| *Nos1* | −2.02 | 0.0004 | ***Myh7*** | −2.29 | 0.0004 | ***Snph*** | −2.74 | 0.0081 |
| *Pax5* | −2.04 | 0.0424 | ***Gm15543*** | −2.30 | 0.0184 | ***Tppp*** | −2.79 | 0.0444 |
| *Pdpr* | −2.04 | 0.0004 | ***Syne1*** | −2.34 | 0.0004 | ***Gm8424*** | −2.80 | 0.0008 |
| *Pfkfb2* | −2.04 | 0.0123 | ***Lrpprc*** | −2.34 | 0.0004 | ***2310057M21Rik*** | −2.91 | 0.0392 |
| *Vegfa* | −2.04 | 0.0004 | ***Mcm2*** | −2.35 | 0.0097 | ***Prkg2*** | −2.96 | 0.0481 |
| *Zfp62* | −2.05 | 0.0155 | ***Pax3*** | −2.38 | 0.0004 | ***Gm47612*** | −2.99 | 0.0021 |
| *Myh13* | −2.05 | 0.0014 | ***Alox15*** | −2.39 | 0.0032 | ***Myh2*** | −3.00 | 0.0004 |
| *Gm42427* | −2.05 | 0.0004 | ***Fxyd3*** | −2.40 | 0.0004 | ***Atm*** | −3.18 | 0.0243 |
| *Perm1* | −2.08 | 0.0004 | ***Ighm*** | −2.44 | 0.0004 | ***Bdh1*** | −3.21 | 0.0004 |
| *Myl3* | −2.08 | 0.0004 | ***Rgs4*** | −2.46 | 0.0004 | ***Dync1li2*** | −3.24 | 0.0004 |
| *Ubap1* | −2.10 | 0.0408 | ***Sesn3*** | −2.47 | 0.0187 | ***Tmem132b*** | −3.29 | 0.0004 |
| *Ppp1r12b* | −2.13 | 0.0004 | ***Dnajb6*** | −2.47 | 0.0249 | ***Rap1gap2*** | −3.32 | 0.0035 |
| *Psd3* | −2.14 | 0.0165 | ***Fbxl3*** | −2.48 | 0.0026 | ***Sp1*** | −3.39 | 0.0081 |
| *Aacs* | −2.15 | 0.0279 | ***Mrs2*** | −2.49 | 0.0008 | ***Lysmd3*** | −3.47 | 0.0058 |
| *9430073C21Rik* | −2.16 | 0.0021 | ***C7*** | −2.49 | 0.0004 | ***Npr3*** | −3.69 | 0.0004 |
| *Smtnl1* | −2.17 | 0.0004 | ***Zfp507*** | −2.52 | 0.0234 | ***Odf3l2*** | −3.98 | 0.0004 |
| *Tnni1* | −2.18 | 0.0004 | ***Tmod1*** | −2.53 | 0.0496 | ***Lncpint*** | −4.04 | 0.0004 |
| *Adgra3* | −2.20 | 0.0026 | ***Mylk4*** | −2.54 | 0.0004 | ***B3galt1*** | −4.08 | 0.0004 |
| *Homer2* | −2.20 | 0.0212 | ***Zfp629*** | −2.58 | 0.0004 | ***Neto2*** | −4.23 | 0.0004 |
| *Myl2* | −2.20 | 0.0004 | ***Sbk3*** | −2.59 | 0.0070 | ***Sall4*** | −4.43 | 0.0023 |
| *Prob1* | −2.21 | 0.0004 | ***Mal*** | −2.64 | 0.0106 | ***Hamp2*** | −5.57 | 0.0165 |
| *Sbk2* | −2.22 | 0.0004 | ***Irx3os*** | −2.68 | 0.0125 |  |  |  |
| *Col22a1* | −2.22 | 0.0398 | ***Ercc6*** | −2.70 | 0.0243 |  |  |  |

FC, fold-change; +, genes found expressed in peritoneum of CHX mice but with no expression detected in control mice (presence/absence genes).
